# Effects of the discontinuation of antihypertensive treatment on neuropsychiatric symptoms and quality of life in nursing home residents with dementia (DANTON): a multicentre, open-label, blinded-outcome, randomised controlled trial

**DOI:** 10.1093/ageing/afae133

**Published:** 2024-07-06

**Authors:** Jonathan M K Bogaerts, Jacobijn Gussekloo, Bianca E M de Jong-Schmit, Saskia Le Cessie, Simon P Mooijaart, Roos C van der Mast, Wilco P Achterberg, Rosalinde K E Poortvliet

**Affiliations:** Department of Public Health and Primary Care, Leiden University Medical Center, Albinusdreef 2, 2333 ZA, Leiden, the Netherlands; LUMC Center for Medicine for Older People, Leiden University Medical Center, Albinusdreef 2, 2333 ZA, Leiden, the Netherlands; Department of Public Health and Primary Care, Leiden University Medical Center, Albinusdreef 2, 2333 ZA, Leiden, the Netherlands; LUMC Center for Medicine for Older People, Leiden University Medical Center, Albinusdreef 2, 2333 ZA, Leiden, the Netherlands; Department of Internal Medicine, Section Gerontology and Geriatrics, Leiden University Medical Center, Albinusdreef 2, 2333 ZA, Leiden, the Netherlands; Department of Public Health and Primary Care, Leiden University Medical Center, Albinusdreef 2, 2333 ZA, Leiden, the Netherlands; LUMC Center for Medicine for Older People, Leiden University Medical Center, Albinusdreef 2, 2333 ZA, Leiden, the Netherlands; LUMC Center for Medicine for Older People, Leiden University Medical Center, Albinusdreef 2, 2333 ZA, Leiden, the Netherlands; Department of Clinical Epidemiology, Leiden University Medical Center, Albinusdreef 2, 2333 ZA, Leiden, the Netherlands; Department of Biomedical Datasciences, Section Medical Statistics, Leiden University Medical Center, Albinusdreef 2, 2333 ZA, Leiden, the Netherlands; LUMC Center for Medicine for Older People, Leiden University Medical Center, Albinusdreef 2, 2333 ZA, Leiden, the Netherlands; Department of Internal Medicine, Section Gerontology and Geriatrics, Leiden University Medical Center, Albinusdreef 2, 2333 ZA, Leiden, the Netherlands; LUMC Center for Medicine for Older People, Leiden University Medical Center, Albinusdreef 2, 2333 ZA, Leiden, the Netherlands; Department of Psychiatry, Leiden University Medical Center, Albinusdreef 2, 2333 ZA, Leiden, the Netherlands; Department of Psychiatry, The Collaborative Antwerp Psychiatric Research Institute (CAPRI), Faculty of Medicine and Health Sciences, University of Antwerp, Campus Drie Eiken, S.033, Universiteitsplein 1, 2610 Wilrijk, Belgium; Department of Public Health and Primary Care, Leiden University Medical Center, Albinusdreef 2, 2333 ZA, Leiden, the Netherlands; LUMC Center for Medicine for Older People, Leiden University Medical Center, Albinusdreef 2, 2333 ZA, Leiden, the Netherlands; University Network for the Care sector South Holland, Leiden University Medical Center, Albinusdreef 2, 2333 ZA, Leiden, the Netherlands; Department of Public Health and Primary Care, Leiden University Medical Center, Albinusdreef 2, 2333 ZA, Leiden, the Netherlands; LUMC Center for Medicine for Older People, Leiden University Medical Center, Albinusdreef 2, 2333 ZA, Leiden, the Netherlands

**Keywords:** antihypertensive treatment, dementia, deprescribing, hypertension, randomised controlled trial, older people

## Abstract

**Background:**

Based on observational studies and randomised controlled trials (RCTs), the benefit–harm balance of antihypertensive treatment in older adults with dementia is unclear.

**Objective:**

To assess whether discontinuing antihypertensive treatment reduces neuropsychiatric symptoms (NPSs) and maintains quality of life (QoL) in nursing home residents with dementia.

**Design:**

Open-label, blinded-outcome RCT. Randomisation 1:1, stratified by nursing home organisation and baseline NPS. Trial registration: NL7365.

**Subjects:**

Dutch long-term care residents with moderate-to-severe dementia and systolic blood pressure (SBP) ≤160 mmHg during antihypertensive treatment. Exclusion criteria included heart failure NYHA-class-III/IV, recent cardiovascular events/procedures or life expectancy <4 months (planned sample size *n* = 492).

**Measurements:**

Co-primary outcomes NPS (Neuropsychiatric Inventory-Nursing Home [NPI-NH]) and QoL (Qualidem) at 16 weeks.

**Results:**

From 9 November 2018 to 4 May 2021, 205 participants (median age 85.8 [IQR 79.6–89.5] years; 79.5% female; median SBP 134 [IQR 123–146] mmHg) were randomised to either antihypertensive treatment discontinuation (*n* = 101) or usual care (*n* = 104). Safety concerns, combined with lacking benefits, prompted the data safety and monitoring board to advice a premature cessation of randomisation. At 16-week follow-up, no significant differences were found between groups for NPI-NH (adjusted mean difference 1.6 [95% CI –2.3 to 5.6]; *P* = 0.42) or Qualidem (adjusted mean difference − 2.5 [95% CI –6.0 to 1.0]; *P* = 0.15). Serious adverse events (SAEs) occurred in 36% (discontinuation) and 24% (usual care) of the participants (adjusted hazard ratio 1.65 [95% CI 0.98–2.79]). All 32-week outcomes favoured usual care.

**Conclusion:**

Halfway through this study, a non-significant increased SAE risk associated with discontinuing antihypertensive treatment was observed, and an associated interim analysis showed that significant worthwhile health gain for discontinuation of antihypertensive treatment was unlikely. This unbeneficial benefit–harm balance shows that discontinuation of antihypertensive treatment in this context does not appear to be either safe or beneficial enough to be recommended in older adults with dementia.

## Key Points

- Observational studies in patients with dementia suggest that antihypertensive treatment may increase the risk for adverse outcomes.
- Previous trials suggest that short-term deprescribing of antihypertensives in older adults without dementia is safe.
- This is the first randomised controlled trial (RCT) that assessed the effects of the discontinuation of antihypertensives in older adults with dementia.
- This prematurely ended RCT found no benefits of discontinuation of antihypertensives and an increased risk of adverse events.
- This negative benefit–harm balance does not support the proactive discontinuation of antihypertensives in older adults with dementia.

## Introduction

Observational population-based cohort studies have shown that low blood pressure (BP) in older adults with physical and cognitive impairments under antihypertensive treatment is associated with an accelerated cognitive decline and an increased all-cause mortality [1–3]. Conversely, landmark randomised controlled trials (RCTs) such as HYVET [4], SPRINT [5] and STEP [6] confirm that starting antihypertensive treatment with tight BP control reduces major cardiovascular events and all-cause mortality in adults 75 years of age or older.

Interestingly, none of these trials included older adults with dementia. Dementia is associated with a gradual drop in systolic blood pressure (SBP) over time [7], and antihypertensive treatment in combination with dementia may produce more frequent side effects, including hypotension, increased anticholinergic burden and falls [8, 9]. Furthermore, observational studies showed that antihypertensive treatment increases the risk for so-called neuropsychiatric symptoms in patients with dementia [10, 11], which are a major factor in reduced quality of life (QoL) [12].

Since the harms of antihypertensive treatment, including side effects and potentially neuropsychiatric symptoms, may outweigh the benefits in the presence of physical and cognitive impairment, older adults with moderate-to-severe dementia residing in long-term care could potentially derive benefits from deprescribing antihypertensive treatment [8, 13]. Outcomes of a handful of RCTs on deprescribing antihypertensive treatment in community-dwelling older adults with short-term follow-up suggest no direct harm [14–16]. However, to date no RCT assessed the benefit–harm balance of deprescribing antihypertensive treatment in older individuals with dementia nor in those residing in long-term care.

Therefore, we investigated the hypothesis that discontinuation of antihypertensive treatment reduces neuropsychiatric symptoms and maintains QoL in older nursing home residents with moderate-to-severe dementia, compared to continuation of antihypertensive treatment.

## Methods

### Study design

The DANTON (Discontinuation of ANtihypertensive Treatment in Older people with dementia living in a Nursing home) study was an open-label, blinded-outcome, RCT conducted in 32 Dutch organisations for long-term care, with follow-ups at 16 (primary endpoint) and 32 weeks (long-term follow-up) after randomisation.

Ethical approval (MEC-Protocol-ID NL65719.058.18) was obtained on 15 October 2018, from the Medical Ethical Committee Leiden-Den Haag-Delft (Leiden, The Netherlands). The full study protocol is accessible online (see online materials: study protocol) [17]. This manuscript followed the consolidated Standards of Reporting Trials (CONSORT) reporting guidelines [18].

The Older Persons Advisory Board Care & Well-being South Holland North has given its full support and endorsement.

### Participants

Long-term care residents with moderate-to-severe dementia living in the nursing homes of 32 long-term care organisations spread throughout the Netherlands, were screened by their treating physician. Long-term care residents with moderate-to-severe dementia according to the Reisberg Global Deterioration Scale [19] (score 5, 6 or 7), who were treated for hypertension with an angiotensin-converting enzyme (ACE) inhibitor, angiotensin-II-receptor blocker, beta blocker, calcium antagonist or diuretic were eligible for inclusion if their SBP was ≤160 mmHg.

Exclusion criteria were an estimated life expectancy of less than 4 months (assessed by the treating physician); heart failure class III–IV according to the functional classification of the New York Heart Association; current angina pectoris; or a recent (<12 months) myocardial infarction, stroke or coronary reperfusion procedure.

After initial eligibility screening by the treating physician, legal representatives of selected residents received a Patient Information Letter explaining the purpose, procedures and possible hazards of the trial. In the Netherlands, legal representation of adults with moderate-to-severe dementia is usually assigned to spouses, siblings or close friends after designation by the district judge. When this is not possible, a volunteer can be appointed as legal representative. In this study, informed consent was provided by the legal representatives of all participating nursing home residents. Legal representatives interested in participation were invited to return the proxy consent form. A research assistant then contacted the legal representative by telephone to ensure the right person had signed the form and to answer any questions.

After confirmation of proxy consent, the resident’s baseline assessment was planned, and both the legal representative and treating physician completed standard questionnaires concerning the resident’s demographic and clinical characteristics.

### Randomisation and masking

After baseline assessment, participants were randomised in a 1:1 ratio to discontinuation of antihypertensive treatment (intervention) or usual care (control). Stratified block randomisation with variable block sizes (2 or 4) and stratification on long-term care organisation and baseline neuropsychiatric symptoms (score ≤ 12 vs. >12 on the total Neuropsychiatric Inventory Nursing Home Version [NPI-NH] [20, 21]) was used to ensure equal distribution within each long-term care organisation and baseline neuropsychiatric symptoms. Allocation concealment was ensured by a central computerised randomisation procedure.

Only treating physicians responsible for the discontinuation intervention were explicitly notified of treatment allocation. Since the study was open-label, participants, legal representatives and nursing home caregivers were not formally blinded to treatment allocation. All research personnel, including a research nurse, an analyst and a chief investigator, were blinded for treatment allocation, except for two data managers due to their roles in randomisation and communication with treating physicians. These data managers were excluded from data collection and analysis.

### Procedures

After notification of allocation to the discontinuation arm, treating physicians were asked to initiate and complete the stepwise, semi-protocolised discontinuation of only those antihypertensive drugs that were prescribed for the indication of hypertension (see Appendix 2, Supplementary Figure S1). Antihypertensive drugs prescribed for other indications, including atrial fibrillation, heart failure or chronic kidney disease, were not stopped. Stepwise discontinuation of antihypertensive treatment was carried on in approximately 6 weeks after randomisation until all antihypertensive drugs were stopped or a maximum SBP of 180 mmHg was reached, based on a previous trial [14]. In case an adverse event and/or abnormal test result occurred, treating physicians could stop the discontinuation protocol or restart antihypertensive treatment when deemed necessary. During discontinuation, SBP and diastolic blood pressure (DBP) were monitored at least weekly. When DBP >120 mmHg, SBP >200 mmHg (>180 mmHg in the presence of diabetes mellitus or a history of cardiovascular diseases) or SBP increased ≥60 mmHg relative to baseline, original antihypertensive treatment was promptly restarted.

Participants allocated to the usual care group continued with their antihypertensive treatment and received the same weekly BP measurements in the first 6 weeks after randomisation.

At baseline, and 16 and 32 weeks after randomisation, a research assistant was scheduled to measure BP during a visit to the nursing home. The duration of the follow-up could exceed 16 and 32 weeks when the treating physician and/or first responsible nurse were temporarily not available. These visits ceased in March 2020 due to the COVID-19 lockdown, and from then until the end of the study, the first responsible nurse or treating physicians completed the BP measurements using the same protocol. Blood pressure was measured twice at rest in a sitting position using a calibrated digital sphygmomanometer on the right arm. The mean of the two measurements was used in analyses.

### Outcomes

The co-primary outcomes were the change from baseline in neuropsychiatric symptoms and QoL at 16 weeks after randomisation. We assessed neuropsychiatric symptoms using the NPI-NH [20, 21] (range 0–144, higher scores indicate more neuropsychiatric symptoms) and QoL using the Qualidem [22, 23] (linearly transformed version with range 0–100, higher scores indicate higher QoL).

The secondary outcome measures were care dependency (Care Dependency Scale [24]; range 15–75, higher scores indicate less dependency), functional status (Katz-15 [25, 26]; range 0–15, higher scores indicate a higher dependence), care-related QoL of (non-professional) caregivers (CarerQoL-7D [27, 28] with VAS; ranges 0–100 and 0–10, respectively, with higher scores indicating higher care-related QoL), cognitive status (7-category Minimum Data Set-Cognitive Performance Scale [29]; categories 0–6, higher scores indicate more severe cognitive impairment), apathy symptoms (abbreviated Apathy Evaluation Scale [30]; range 10–40, higher scores indicate more apathy symptoms) and the presence of delirium (we used the short version of the Confusion Assessment Method [CAM] [31] instead of the Nursing Home Confusion Assessment Method [NH-CAM] [32]; categories ‘no delirium’, ‘probably delirium’ and ‘delirium’).

Cognitive status, neuropsychiatric symptoms and delirium status were evaluated in interviews with the participant’s first responsible nurse using the aforementioned instruments. Other outcomes were evaluated with standardised written questionnaires. The care-related QoL of (non-professional) caregivers was part of a questionnaire package completed by the legal representative. For evaluation of (psychotropic) medication use over time, medication overviews were collected. Additionally, the number of falls from 16 weeks before baseline to the last follow-up measurement was extracted from electronic medical records.

Additional outcomes were the (professional) Caregiver Distress Scale of the NPI-NH (range 0–60, higher scores indicate more distress) and discomfort (the Discomfort Scale for Dementia of the Alzheimer’s Type [DS-DAT][33]; range 0–27, higher scores indicate more discomfort).

The presence of orthostatic hypotension, neuropsychiatric symptoms registered in the electronic medical records and psychosocial interventions started for neuropsychiatric symptoms will not be reported in this publication since these outcomes have not been assessed due to logistic problems (text mining strategies have not been carried out) and the validity of the measurement has not yet been ascertained.

Long-term effects on co-primary and secondary outcomes were analysed over 32 weeks.

Cost-effectiveness analysis was only done when there was a beneficial effect of the intervention.

### Serious adverse events

Serious adverse events (SAEs) were defined as death of any cause, clinical diagnoses of life-threatening illness, myocardial infarction, stroke, transient ischaemic attack or any non-elective hospitalisation between randomisation and the end of follow-up. A new SAE was reported within 7 days of first knowledge to the Data Safety and Monitoring Board (DSMB) and the medical ethics committee.

After the trial ended, all information on collected SAEs was presented to an independent panel of five physicians (three elderly care physicians and two general practitioners) to individually label each SAE as ‘cardiovascular’, ‘non-cardiovascular’ or ‘not possible to determine’.

### Sample size

Based on an assumed standard deviation (SD) of 11 and assuming a dropout rate of 35%, we aimed for a total sample size of 492 participants (246 per randomisation group), which would provide 90% power using a two-sided alpha of 0.05 for detection of a mean difference in change of the NPI-NH score of four points (considered as a relevant change by the developers and used in previous trials as a clinically relevant change) [34, 35] between baseline and follow-up at 16 weeks after randomisation. This sample size can detect a mean difference between groups in linearly transformed Qualidem scores of seven points (corresponding to 10% of the median score of a comparable population [36]), with an assumed SD of 13. The study was considered positive if a significant difference (*P*-value <0.05) of, respectively, four and seven points was achieved for both co-primary outcomes.

### Analytical methods

A blinded-outcome analysis was used. Baseline characteristics are described per randomisation group as means (SD), medians (interquartile range [IQR]) or numbers (percentage), where appropriate. All analyses were carried out according to the intention-to-treat principle. The mean differences in the co-primary outcomes were calculated using a linear mixed regression model that included the difference between follow-up score and baseline score as the dependent variable, the baseline score of the investigated outcome as a co-variate and randomisation group as a factor (Analysis of covariance (ANCOVA) approach). As randomisation was stratified, the baseline total NPI-NH score (binary, ≤12 vs. >12) was added to the model as a fixed effect and long-term care organisation was added as a random effect with an assumed normal distribution.

Secondary outcomes were comparably analysed, using generalised linear mixed models. Differences in psychotropic drugs were analysed by binary logistic regression (by type). For cognitive status and presence of delirium, ordinal logistic regression was used (cumulative logit model). Differences in numbers of falls were analysed by using Poisson regression analyses with the same fixed and random effects.

The incidences of SAEs are presented as Kaplan–Meier plots by randomisation group for the time to the first occurrence of both all-cause SAE and all-cause mortality. In addition, we performed time-to-event comparisons, with censoring for competing risks of non-cardiovascular SAEs and non-cardiovascular death. Supplementary to our statistical analysis plan (fully accessible online; see online materials: statistical analysis plan) [37], Cox proportional-hazards models were used to obtain hazard ratios (HRs) to compare the randomisation groups for all-cause SAE and all-cause mortality, adjusted for age, sex, history of cardiovascular disease and baseline NPI-NH score (binary, ≤12 vs. >12) as a fixed effect and long-term care organisation as a random effect (with Gaussian frailty). The proportional hazards assumption was checked using plots of the Schoenfeld residuals versus log(time) and the corresponding test for non-proportional hazards.

#### Subgroup analyses

We further performed two planned subgroup analyses, one defined by stratification by baseline NPI-NH (score ≤ 12 vs. >12), one defined by stratification by the linearly transformed baseline Qualidem (score <70 vs. ≥70). Post hoc, we added a subgroup analysis defined by stratification by baseline SBP (<134 mmHg vs. ≥134 mmHg).

In Appendix 1 we describe the per-protocol analyses, a sensitivity analysis with additional adjustment for relevant imbalances between randomisation groups and the analyses of the HRs for first cardiovascular events.

Analyses were performed with SPSS software (version 25.0; IBM Corp). The analyses of the SAEs were performed using R version 4.1.2 with software packages rms, survival and Hmisc. This trial is registered with the Netherlands Trial Register (ID-NL7365).

The installed independent DSMB was composed of a cardiologist, a neurologist and a statistician. The formalised role of the DSMB was to monitor the safety of participants of the trial. The DSMB was asked to base their advice on their clinical perspective, and there were no formal stopping rules (for charter, see online materials: DSMB charter). The DSMB would meet after the first 50 participants’ completion of the 16-week follow-up measurement and subsequently after every 100 additional participants. In the closed part of the meeting, the DSMB had access to an unblinded report including all BP outcomes and information on all SAEs. In addition to the BP and SAE data as defined in the pre-study charter, the DSMB requested and received unblinded access to the co-primary outcomes at 16-week follow-up. The DSMB used these outcome data to judge the safety against efficacy benefits. The statistician of the DSMB could perform a conditional power analysis when deemed necessary.

## Results

Between 9 November 2018, and 4 May 2021, we screened 6,252 long-term care nursing home residents for inclusion and exclusion criteria. We received initial informed consent for 316 of 1,248 eligible participants. On reviewing the seventh unblinded report that included data from 194 randomised participants, of whom 136 had completed the 16-week follow-up measurement, the DSMB advised on 15 May 2021 that randomisation of new participants should be stopped. This was deemed necessary based on the safety concerns, and that the trial was not realistically expected to show a beneficial effect on the co-primary outcomes. Based on this advice and after discussion with the Medical Ethical Committee, it was decided that the follow-up would continue for all randomised participants until their next follow-up measurement.

**Figure 1:**
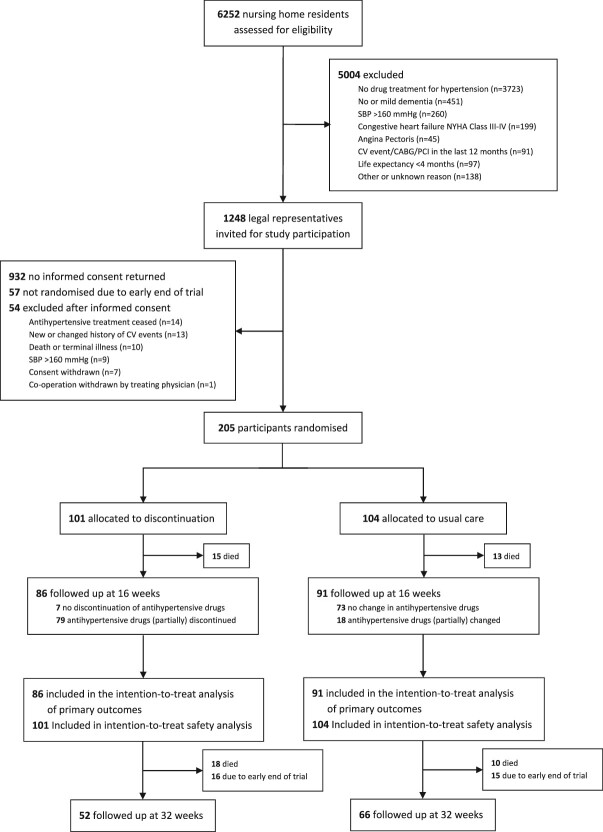
Flow of participants through study. SBP = systolic blood pressure; NYHA = New York Heart Association; CV = cardiovascular; CABG = Coronary artery bypass grafting; PCI = Percutaneous coronary intervention.

With 57 participants not randomised due to the study’s premature cessation of randomisation and 54 participants not meeting the final eligibility criteria, we randomised a total of 205 participants to either the discontinuation (*n* = 101) and usual care (*n* = 104) group (Figure 1). Of the 205 participants, 177 completed the 16-week follow-up. The study ended on 13 October 2021, with 118 of 177 participants completing the 32-week follow-up. Most data on primary outcomes were complete (only 0.2% missing datapoints in the Qualidem questionnaire). The percentages of missing data on secondary outcomes ranged from 0.0% to 17.4%.

 At baseline, most demographics, clinical characteristics and prescribed drugs were well balanced between groups (Table 1; Appendix 2, Supplementary Table S1). The median age of participants was 85.8 (IQR 79.6–89.5) years, 163 (79.5%) of whom were female. Median SBP was 134 (IQR 123–146) mmHg and median DBP 72 (IQR 64–80) mmHg. The median number of antihypertensive drugs per participant was 2 (IQR 1–2), and 127 (62.0%) participants had a history of cardiovascular disease.

**Table 1:** Baseline demographics and clinical characteristics of participants

		Discontinuation (*n* = 101)	Usual care (*n* = 104)
Age in years, median (IQR)		85.3 (80.1–88.4)	86.6 (79.2–89.8)
Female, *n* (%)		77 (76.2)	86 (82.7)
SBP in mmHg, median (IQR)		134 (122–147)	133 (124–145)
DBP in mmHg, median (IQR)		72 (62–81)	73 (65–80)
History of cardiovascular disease, *n* (%)^a^		59 (58.4)	68 (65.4)
Diabetes mellitus, *n* (%)^b^		25 (24.8)	29 (27.9)
Number of antihypertensive drugs, median (IQR)		2 (1–2)	1.5 (1–2)
Antihypertensive drugs, *n* (%)			
	ACE inhibitor	42 (41.6)	38 (36.5)
	Angiotensin receptor blocker	24 (23.8)	20 (19.2)
	Beta blocker	49 (48.5)	53 (51.0)
	Calcium channel blocker	26 (25.7)	29 (27.9)
	Loop diuretic	15 (14.9)	15 (14.4)
	Potassium-sparing diuretic	5 (5.0)	4 (3.8)
	Thiazide diuretic	18 (17.8)	17 (16.3)
Number of psychotropic drugs, median (IQR)		1 (0–2)	1 (0–2)
Other drugs, *n* (%)			
	Anticoagulant	26 (25.7)	18 (17.3)
	Antiplatelet	32 (31.7)	40 (38.5)
	Statin	29 (28.7)	19 (18.3)
Number of ATC-coded drugs, median (IQR)		10 (7–12)	10 (7–11.5)
Dementia type, *n* (%)^c^			
	Alzheimer	45 (44.6)	37 (35.6)
	Vascular	18 (17.8)	14 (13.5)
	Frontotemporal	2 (2.0)	2 (1.9)
	Lewy Body	1 (1.0)	0
	Mixed or Other	19 (18.8)	20 (19.2)
	Unknown	16 (15.8)	31 (29.8)
Dementia stage, *n* (%)			
	Reisberg GDS Stage 5	45 (44.6)	41 (39.4)
	Reisberg GDS Stage 6	50 (49.5)	52 (50.0)
	Reisberg GDS Stage 7	6 (5.9)	11 (10.6)
Neuropsychiatric symptoms	NPI-NH, median (IQR)	15 (2–31)	12 (3–24)
Dementia-specific QoL	Linearly transformed Qualidem, median (IQR)	64.8 (51.9–81.5)	70.4 (55.6–79.6)
Professional caregiver distress	NPI-NH subscale	6 (1–14)	5 (1–12)
Dementia-related discomfort	DS-DAT, median (IQR)	6 (4–7)	5.5 (4–7)
Apathy symptoms	AES-10, median (IQR)	28 (24–33)	30 (24–35)
Care dependency	CDS, median (IQR)	40 (32–51)	40 (30–51)
Functional status	Katz-15, median (IQR)	12 (10–14)	12 (10–14)
Care-related QoL of the legal representative	CarerQoL-7D index, median (IQR)	85.5 (78.0–92.9)	87.8 (77.6–92.5)
Care-related QoL of the legal representative	CarerQoL-7D-VAS, median (IQR)	8 (7–8)	8 (7–8)
Cognitive status (MDS-CPS), *n* (%)			
	Score 0–2	30 (29.7)	36 (34.6)
	Score 3–4	29 (28.7)	22 (21.2)
	Score 5–6	42 (41.6)	46 (44.2)
Presence of delirium (Short CAM), *n* (%)			
	No delirium	71 (70.3)	77 (74.0)
	Probably delirium	18 (17.8)	18 (17.3)
	Delirium	12 (11.9)	9 (8.7)
Number of falls in the preceding 112 days		0 (0–1)	0 (0–1)

ACE, angiotensin-converting enzyme; AES-10, abbreviated Apathy Evaluation Scale; ATC, Anatomical Therapeutic Chemical; CAM, Confusion Assessment Method; CarerQoL, care-related quality-of-life of caregivers; CDS, Care Dependency Scale; DBP, diastolic blood pressure; DS-DAT, Discomfort scale for patients with dementia of the Alzheimer type; GDS, Global Deterioration Scale. IQR, interquartile range; MDS-CPS, The Minimum Data Set Cognitive Performance Scale; NPI-NH, Neuropsychiatric Inventory Nursing Home; QoL, quality of life; SBP, systolic blood pressure; VAS, visual analogue scale.

^a^Defined as a history of myocardial infarction, angina pectoris, stroke, transient ischemic attack, heart failure, peripheral arterial disease, (coronary) bypass surgery, reperfusion procedure or atrial fibrillation.

^b^As reported in the electronic medical record and/or under treatment with blood glucose–lowering drugs (including insulin).

^c^As reported by the treating physician.

Table 2 presents the implementation of the intervention. At 16-week follow-up, an average of 1.3 (SD 0.9) antihypertensive drugs were withdrawn in the discontinuation group compared with 0.1 (SD 0.4) in the usual care group (adjusted mean difference − 1.2 [95% CI –1.4to −1.0]; *P* < 0.001), a difference maintained (−1.0 [95% CI –1.2 to −0.8]; *P* < 0.001) at 32 weeks. At 32 weeks, antihypertensive treatment was restarted in 5.8% of the discontinuation group. At 16 weeks, the adjusted mean difference in change of SBP was 11.0 mmHg (95% CI 5.6 to 16.4; *P* < 0.001); at 32 weeks, this was 4.9 mmHg (95% CI −0.8 to 10.6; *P* = 0.089). The adjusted mean difference in DBP was 4.6 mmHg (95% CI 1.1 to 8.1; *P* = 0.010) at 16-week follow-up and 3.3 mmHg (95% CI –0.5 to 7.2; *P* = 0.088) at 32-week follow-up. Prescription details for individual antihypertensive drugs are given in Appendix 2 and Supplementary Table S2.

**Table 2:** Implementation of intervention at 16 and 32 weeks after randomisation

	Mean change between baseline and follow-up (SD)				Mean between-group difference (95% CI)		
	*n*	Discontinuation	*n*	Usual care	Unadjusted	Adjusted^a^	*P*-value^a^
Antihypertensive drugs^b^							
16 weeks	86	−1.3 (0.9)	91	−0.1 (0.4)	−1.2 (−1.4 to −1.0)	−1.2 (−1.4 to −1.0)	<0.001
32 weeks	50	−1.2 (0.8)	65	−0.2 (0.5)	−1.0 (−1.3 to −0.8)	−1.0 (−1.2 to −0.8)	<0.001
SBP in mmHg^b^							
16 weeks	86	10.2 (24.3)	91	−0.5 (17.2)	10.8 (4.5 to 17.0)	11.0 (5.6 to 16.4)	<0.001
32 weeks	52	6.6 (18.3)	65	1.7 (19.0)	4.9 (−2.0 to 11.8)	4.9 (−0.8 to 10.6)	0.089
DBP in mmHg^b^							
16 weeks	86	7.5 (14.8)	91	2.6 (12.0)	4.9 (0.9 to 8.9)	4.6 (1.1 to 8.1)	0.010
32 weeks	52	9.3 (13.6)	65	5.3 (11.7)	3.9 (−0.7 to 8.6)	3.3 (−0.5 to 7.2)	0.088

CI, confidence interval; DBP, diastolic blood pressure; SBP, systolic blood pressure; SD, standard deviation.

^a^Adjusted for the pre-specified factors: baseline value of the investigated outcome, baseline Neuropsychiatric Inventory Nursing Home score (binary, ≤12 vs. >12) and long-term care organisation.

^b^Positive mean change corresponds with an increase from baseline, negative mean change corresponds with a decrease from baseline. Positive between-group difference corresponds with a higher increase in the discontinuation group, while a negative between-group difference corresponds with a higher decrease in the discontinuation group.

The co-primary outcomes of the study are depicted in Table 3. At 16 weeks, changes in NPI-NH (adjusted mean difference 1.6 [95% CI –2.3 to 5.6]; *P* = 0.42) and Qualidem (adjusted mean difference − 2.5 [95% CI –6.0 to 1.0]; *P* = 0.15) scores did not differ statistically between groups.

**Table 3:** Primary outcomes at 16 and 32 weeks after randomisation by intention-to-treat analysis

	Mean change between baseline and follow-up (SD)				Mean between-group difference (95% CI)		
	*n*	Discontinuation	*n*	Usual care	Unadjusted	Adjusted^a^	*P*-value^a^
NPI-NH^b^							
16 weeks	86	−2.6 (15.3)	91	−2.8 (14.2)	0.2 (−4.2 to 4.5)	1.6 (−2.3 to 5.6)	0.42
32 weeks	52	−1.2 (15.6)	66	−6.4 (13.3)	5.3 (0.0 to 10.5)	6.2 (1.9 to 10.6)	0.0050
Qualidem (LT)^c^							
16 weeks	86	0.6 (13.6)	91	2.0 (12.2)	−1.4 (−5.2 to 2.5)	−2.5 (−6.0 to 1.0)	0.15
32 weeks	52	4.1 (15.4)	65	5.2 (13.2)	−1.2 (−6.4 to 4.1)	−3.5 (−8.1 to 1.1)	0.13

Higher scores on the NPI-NH indicate more neuropsychiatric symptoms, higher scores on the Qualidem indicate higher dementia-specific QoL. CI, confidence interval; LT, linearly transformed; NPI-NH, Neuropsychiatric Inventory Nursing Home; SD, standard deviation.

^a^Adjusted for prespecified factors: baseline value of the investigated outcome, baseline NPI-NH score (binary, ≤12 vs >12) and long-term care organisation.

^b^Positive mean change corresponds with a deterioration from baseline, negative mean change corresponds with an improvement from baseline.

^c^Positive mean change corresponds with an improvement from baseline, negative mean change corresponds with a deterioration from baseline.

At 32 weeks, the control group had fewer neuropsychiatric symptoms, with an adjusted mean difference in change from baseline of 6.2 points (95% CI 1.9 to 10.6; *P* = 0.0050), while the change in Qualidem scores was not statistically different (adjusted mean difference − 3.5 [95% CI –8.1 to 1.1]; *P* = 0.13).

All secondary outcomes were comparable between groups at 16 weeks (Table 4). At 32 weeks, the secondary outcomes showed no difference or a disadvantage for the discontinuation group (Table 4; for a detailed prescription of psychotropic use, see Appendix 2, Supplementary Tables S3 and S4). A negative change in professional caregiver distress was noted for the discontinuation group (adjusted mean difference 2.7 [95% CI 0.8–4.7]; *P* = 0.006). Other measures related to wellbeing, such as the between-group differences in DS-DAT score, indicated higher discomfort in the discontinuation group at 32 weeks. Discontinuing antihypertensive treatment was associated with a higher risk of delirium (adjusted odds ratio 3.07 [95% CI 1.23–7.66]; *P* = 0.017) and falling (adjusted mean ratio 2.21 [95% CI 1.56–3.13]; *P* < 0.001).

**Table 4:** Secondary and additional outcomes at 16 and 32 weeks after randomisation by intention-to-treat analysis

	Mean change between baseline and follow-up (SD)				Mean between-group difference (95% CI)		OR (95% CI)		MR (95% CI)		
	*n*	Discontinuation	*n*	Usual care	Unadjusted	Adjusted^a^	Unadjusted	Adjusted^a^	Unadjusted	Adjusted^a^	*P*- value^a^
NPI-NH Caregiver distress^b^											
16 weeks	86	−0.9 (6.8)	91	−1.7 (6.0)	0.8 (−1.1 to 2.7)	1.4 (−0.3 to 3.0)	–	–	–	–	0.11
32 weeks	52	−0.6 (7.0)	66	−2.8 (6.6)	2.3 (−0.2 to 4.7)	2.7 (0.8 to 4.7)	–	–	–	–	0.0060
Psychotropic drugs^b^											
16 weeks	86	0.05 (0.5)	91	0.03 (0.3)	0.01 (−0.11 to 0.14)	0.01 (−0.11 to 0.14)	–	–	–	–	0.84
32 weeks	50	0.10 (0.5)	65	0.05 (0.4)	0.05 (−0.11 to 0.22)	0.05 (−0.10 to 0.21)	–	–	–	–	0.49
DS-DAT^b^											
16 weeks	86	−0.8 (2.9)	91	−1.0 (2.5)	0.2 (−0.6 to 1.0)	0.4 (−0.2 to 1.0)	–	–	–	–	0.16
32 weeks	52	−2.1 (3.1)	66	−2.0 (3.2)	−0.1 (−1.3 to 1.0)	0.7 (0.0 to 1.3)	–	–	–	–	0.046
AES-10^b^											
16 weeks	84	0.4 (8.2)	84	0.7 (6.1)	−0.3 (−2.5 to 1.9)	−1.1 (−3.0 to 0.8)	–	–	–	–	0.24
32 weeks	48	1.6 (6.9)	61	2.0 (6.9)	−0.4 (−3.1 to 2.2)	−0.8 (−3.2 to 1.6)	–	–	–	–	0.51
CDS^c^											
16 weeks	86	−2.1 (9.3)	91	−1.3 (8.7)	−0.8 (−3.5 to 1.8)	−0.2 (−2.8 to 2.4)	–	–	–	–	0.87
32 weeks	52	−5.2 (11.7)	65	−2.8 (9.2)	−2.3 (−6.1 to 1.5)	−1.4 (−5.1 to 2.3)	–	–	–	–	0.47
Katz-15^b^											
16 weeks	86	0.5 (2.2)	91	0.1 (1.7)	0.4 (−0.2 to 1.0)	0.1 (−0.4 to 0.6)	–	–	–	–	0.63
32 weeks	52	1.0 (2.4)	65	0.6 (1.4)	0.4 (−0.3 to 1.2)	0.3 (−0.4 to 1.0)	–	–	–	–	0.41
CarerQoL-7D index^c^											
16 weeks	66	−1.5 (8.3)	75	−1.6 (11.7)	0.1 (−3.3 to 3.6)	0.3 (−3.0 to 3.6)	–	–	–	–	0.86
32 weeks	35	0.7 (10.5)	52	−0.5 (11.3)	1.1 (−3.6 to 5.9)	0.4 (−3.9 to 4.8)	–	–	–	–	0.84
CarerQoL-7D-VAS^c^											
16 weeks	65	−0.4 (1.2)	77	−0.2 (1.0)	−0.2 (−0.6 to 0.2)	−0.2 (−0.6 to 0.2)	–	–	–	–	0.32
32 weeks	36	−0.5 (1.3)	55	−0.1 (1.1)	−0.4 (−0.9 to 0.1)	−0.4 (−0.9 to 0.1)	–	–	–	–	0.11
MDS-CPS^d^											
16 weeks		–		–	–	–	1.10 (0.65 to 1.87)	1.12 (0.65 to 1.92)	–	–	0.69
32 weeks		–		–	–	–	0.66 (0.34 to 1.26)	0.84 (0.43 to 1.66)	–	–	0.84
Short CAM^e^											
16 weeks		–		–	–	–	1.70 (0.85 to 3.41)	1.66 (0.79 to 3.48)	–	–	0.18
32 weeks		–		–	–	–	3.00 (1.25 to 7.19)	3.07 (1.23 to 7.66)	–	–	0.017
Falls^f^											
16 weeks		–		–	–	–	–	–	1.02 (0.76 to 1.38)	0.95 (0.70 to 1.29)	0.73
32 weeks		–		–	–	–	–	–	2.09 (1.50 to 2.92)	2.21 (1.56 to 3.13)	<0.001

AES-10, abbreviated Apathy Evaluation Scale; CAM, Confusion Assessment Method; CarerQoL, care-related quality-of-life of caregivers; CDS, Care Dependency Scale; CI, confidence interval; DS-DAT, Discomfort scale for patients with dementia of the Alzheimer type; MDS-CPS, The Minimum Data Set Cognitive Performance Scale; MR, mean ratio; NPI-NH, Neuropsychiatric Inventory Nursing Home; OR, odds ratio; SD, standard deviation; VAS, visual analogue scale.

^a^Adjusted for prespecified factors: baseline value of the investigated outcome, baseline NPI-NH score (binary, ≤12 vs >12) and long-term care organisation.

^b^A positive mean change corresponds with a deterioration from baseline; a negative mean change corresponds with an improvement from baseline.

^c^A positive mean change corresponds with an improvement from baseline; a negative mean change corresponds with a deterioration from baseline.

^d^An OR < 1.00 corresponds with a higher odds on faster cognitive decline in the usual care group; an OR > 1.00 corresponds with a higher odds on faster cognitive decline in the discontinuation group.

^e^An OR < 1.00 corresponds with a higher odds on a delirium in the usual care group; an OR > 1.00 corresponds with a higher odds on a delirium in the discontinuation group.

^f^An MR <1.00 corresponds with a higher ratio of falls in the usual care group; an MR >1.00 corresponds with a higher ratio of falls in the discontinuation group.

**Figure 2:**
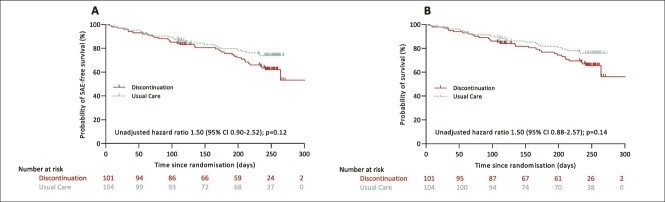
Unadjusted Kaplan-Meier estimates of the event-free (A) and overall (B) survival from randomisation up to day 300. SAE = Serious adverse event.

All SAEs between randomisation and 32-week follow-up were recorded. In total, 63 SAEs were reported in 61 participants, comprising 37 SAEs in 36 (35.6%) discontinuation group participants after a median of 135 (IQR 66–209) days and 26 SAEs in 25 (24.0%) usual care group participants after a median of 103 (IQR 54–171) days (Table 5; for detailed information on participants with a reported SAE, see Appendix 2 and Supplementary Tables S5 and S6). At 16 weeks, the adjusted HR for first all-cause SAE were 1.38 (95% CI 0.67–2.82; *P* = 0.38) and for all-cause mortality 1.55 (95% CI 0.73–3.31; *P* = 0.25). At 32 weeks, both the risk of first all-cause SAE (adjusted HR 1.65 [95% CI 0.98–2.79; *P* = 0.062]) and all-cause mortality (adjusted HR 1.65 [95% CI 0.95–2.85; *P* = 0.074]) appeared elevated in the discontinuation group (Figure 2).

**Table 5:** Serious adverse events

		Discontinuation (*n* = 101)	Usual care (*n* = 104)
**Participants with reported SAE** ^**^a^**^ **(%)**		36 (35.6)	25 (24.0)
	Cardiovascular SAE, *n* (%)	9 (8.9)	5 (4.8)
	Non-cardiovascular SAE, *n* (%)	26 (25.7)	18 (17.3)
	Not possible to determine, *n* (%)	1 (1.0)	2 (1.9)
	Time to SAE in days, median (IQR)	135 (66–209)	103 (54–171)
**Death, *n* (%)**		33 (32.7)	23 (22.1)
	Cardiovascular, *n* (%)	7 (6.9)	5 (4.8)
	Non-cardiovascular, *n* (%)	25 (24.8)	16 (15.4)
	Not possible to determine, *n* (%)	1 (1.0)	2 (1.9)
	Time to death in days, median (IQR)	134 (68–208)	111 (56–173)
**Transient ischaemic attack, *n* (%)**		0	1 (1.0)
**Stroke, *n* (%)**		3 (3.0)	1^b^(1.0)
**Myocardial infarction, *n* (%)**		2 (2.0)	0
**Hospitalisation, *n* (%)**		3 (3.0)	1 (1.0)

In total, 63 SAEs were reported in 61 participants, comprising 37 SAEs in 36 (35.6%) discontinuation group participants and 26 SAEs in 25 (24.0%) usual care group participants. IQR, interquartile range; SAE, serious adverse event.

^a^For the two participants with multiple SAEs, the first SAE is included.

^b^One lethal SAE was described as a COVID-19 infection or a secondary aspiration pneumonia after a preceding stroke.

 Additional subgroup analyses stratified by baseline NPI-NH, Qualidem and SBP indicated that the differences in co-primary outcomes between randomisation groups showed greater disadvantage for participants with higher neuropsychiatric symptoms, lower QoL and higher SBP at baseline (Table 6). Subgroup analyses yielded roughly similar HRs for incidences of all-cause SAE and all-cause mortality in all subgroups, but more prominent in the subgroup with lower SBP at baseline (Table 6).

**Table 6:** Primary and safety outcomes at 16 and 32 weeks of follow-up stratified by neuropsychiatric symptoms, QoL and systolic blood pressure at baseline by intention-to-treat analysis

		Mean between-group differences of change between baseline and follow-up (95% CI)						Hazard ratio (95% CI)					
		Outcome NPI-NH			Outcome Qualidem (LT)			All-cause SAE			All-cause mortality		
		*n*	Adjusted^a^	*P* ^a^	*n*	Adjusted^a^	*P* ^a^	*n*	Unadjusted	*P*	*n*	Unadjusted	*P*
**16 weeks**													
Overall													
		177	1.6 (−2.3 to 5.6)	0.42	177	−2.5 (−6.0 to 1.0)	0.15	205	1.27 (0.63 to 2.57)	0.51	205	1.39 (0.66 to 2.95)	0.39
NPI-NH													
	≤12	88	−1.9 (−5.3 to 1.6)	0.29	88	2.1 (−2.2 to 6.4)	0.34	103	1.30 (0.47 to 3.59)	0.61	103	1.52 (0.53 to 4.37)	0.44
	>12	89	5.7 (−1.4 to 12.8)	0.12	89	−7.2 (−12.4 to −2.1)	0.0070	102	1.23 (0.46 to 3.30)	0.68	102	1.28 (0.44 to 3.68)	0.65
Qualidem (LT)													
	<70	90	3.6 (−3.5 to 10.8)	0.32	90	−4.1 (−10.0 to 1.7)	0.16	102	1.07 (0.39 to 2.96)	0.89	102	1.32 (0.42 to 4.15)	0.64
	≥70	87	−0.7 (−4.1 to 2.6)	0.68	87	−0.5 (−4.2 to 3.2)	0.79	103	1.52 (0.57 to 4.09)	0.40	103	1.52 (0.57 to 4.09)	0.40
SBP													
	<134	93	−3.5 (−8.9 to 1.9)	0.20	93	1.2 (−3.4 to 5.8)	0.60	106	3.24 (0.88 to 11.98)	0.078	106	3.24 (0.88 to 11.98)	0.078
	≥134	84	7.4 (1.7 to 13.2)	0.011	84	−6.6 (−12.0 to −1.3)	0.015	99	0.72 (0.29 to 1.79)	0.48	99	0.77 (0.29 to 2.07)	0.61
**32 weeks**													
Overall													
		118	6.2 (1.9 to 10.6)	0.0050	117	−3.5 (−8.1 to 1.1)	0.13	205	1.50 (0.90 to 2.52)	0.12	205	1.50 (0.88 to 2.57)	0.14
NPI-NH													
	≤12	56	3.3 (−0.7 to 7.3)	0.10	55	−1.2 (−7.3 to 5.0)	0.71	103	1.27 (0.58 to 2.79)	0.55	103	1.29 (0.57 to 2.92)	0.54
	>12	62	8.9 (1.3 to 16.5)	0.022	62	−5.4 (−12.3 to 1.5)	0.12	102	1.67 (0.84 to 3.32)	0.15	102	1.65 (0.81 to 3.38)	0.17
Qualidem (LT)													
	<70	63	9.9 (2.5 to 17.4)	0.010	62	−2.8 (−9.8 to 4.2)	0.42	102	1.55 (0.78 to 3.10)	0.21	102	1.70 (0.81 to 3.57)	0.16
	≥70	55	2.0 (−2.1 to 6.2)	0.33	55	−3.7 (−9.3 to 1.9)	0.19	103	1.45 (0.67 to 3.14)	0.34	103	1.34 (0.61 to 2.93)	0.47
SBP													
	<134	62	6.7 (0.4 to 13.0)	0.038	61	−1.0 (−7.5 to 5.4)	0.75	106	2.05 (1.00 to 4.20)	0.050	106	1.93 (0.94 to 3.99)	0.074
	≥134	56	6.0 (−0.5 to 12.5)	0.069	56	−5.6 (−12.6 to 1.3)	0.11	99	1.11 (0.53 to 2.33)	0.79	99	1.16 (0.52 to 2.58)	0.73

CI, confidence interval; LT, linearly transformed; NPI-NH, Neuropsychiatric Inventory Nursing Home; SAE, serious adverse event. SBP, systolic blood pressure.

^a^Adjusted for prespecified factors: baseline value of the investigated outcome, baseline NPI-NH score (binary, ≤12 vs >12) and long-term care organisation.

## Discussion

In the present multicentre open-label RCT discontinuation of antihypertensive treatment did not improve neuropsychiatric symptoms or QoL of older people with moderate-to-severe dementia at 16- and 32-week follow-up. Since there was an increase in adverse events, this RCT shows a negative benefit–harm balance of the discontinuation of antihypertensive treatment in older people with moderate-to-severe dementia.

This study is the first to explore the benefit–harm balance of the discontinuation of antihypertensive treatment in older adults with moderate-to-severe dementia based on a nationwide multicentre RCT in the nursing home setting. We successfully investigated this important research question despite a worldwide pandemic. As the outcomes of the study were derived from a wide variety of sources that all individually indicated either no benefit or a negative effect of the discontinuation of antihypertensive treatment, we are confident that our results are internally valid.

The premature cessation of randomisation of this study resulted in a smaller sample size than anticipated, which may have diluted the intervention and the outcomes. However, the confidence intervals of the analyses imply that a meaningful benefit would be unlikely if the trial would be completed as planned. It could be seen as another limitation that broader assessments of blood samples, echocardiograms and neurovascular imaging scans are lacking. Although these data would have permitted in-depth physiological and, potentially, a causal interpretation of our results, the burden of these tests conflicted with the comfort-oriented goals of care in patients with moderate-to-severe dementia. A double-blind placebo-controlled design would have been optimal as was recently published by Etherton-Beer *et al* [38]. We considered this as unfeasible in the daily practice of a Dutch nursing home setting. To study potential differences in the effects of discontinuation between classes of antihypertensive medication, the subgroups became too small.

To our knowledge, this study is the first open-label RCT to assess the effects of discontinuing antihypertensive treatment in older adults with dementia. The blueprint for the present trial, the DANTE study [14], which was conducted in a population of older people with milder cognitive deficits, showed no benefit but also no harm associated with discontinuing antihypertensive treatment at 4-month follow-up. Likewise, a meta-analysis by Reeve *et al* [15]., which included studies on the withdrawal of antihypertensive drugs used for hypertension or primary cardiovascular disease prevention in people older than 50 years, concluded that there was no effect of deprescribing antihypertensive treatment on all-cause mortality or myocardial infarction, albeit with limited evidence. The OPTIMISE trial [16], the only RCT on deprescribing antihypertensive treatment published since that meta-analysis, showed that reducing antihypertensive treatment in octogenarians with SBP <150 mmHg was non-inferior to usual care at 12 weeks regarding SBP and SAE incidence. Compared to the DANTE study [14] and OPTIMISE trial[16], participants in our study had lower cognitive functioning and more co-morbidities. Furthermore, the increment in SBP in the DANTON study was slightly higher: 11.0 mmHg versus 7.4 mmHg in the DANTE study [14] and 3.4 mmHg in the OPTIMISE Trial [16], and our follow-up was longer.

We offer several possible explanations for the negative benefit–harm balance of the discontinuation of antihypertensive treatment in the current trial. One hypothesis is that discontinuation of antihypertensive treatment disrupts a fragile homeostasis in these vulnerable older patients with dementia. A comparable mechanism was observed in the discontinuation of cholinesterase inhibitors and memantine. Questioning the benefit–harm balance of these drugs in dementia, a meta-analysis showed that discontinuation of cholinesterase inhibitors and memantine resulted in a worse cognitive, neuropsychiatric and functional status [39]. Another explanation could be that discontinuing antihypertensive treatment progressively leads to a worsening of subclinical cerebrovascular disease or undiagnosed heart failure, the latter a condition frequently missed in the nursing home setting [40]. Another possibility is that discontinuation of antihypertensive treatment attenuates other beneficial effects of these drugs such as antioxidant activity and reduced production of pro-inflammatory proteins, resulting in more endothelial dysfunction and vascular inflammation [41], eventually leading to more SAEs. Additionally, it cannot be ruled out that the higher incidence of SAEs in the intervention group is a chance finding.

To conclude, halfway through this study, a non-significant increase in the risk of SAEs associated with discontinuing antihypertensive treatment was observed, and an associated interim analysis showed that significant worthwhile health gain for discontinuation of antihypertensive treatment was unlikely. This unbeneficial balance between benefits and harms shows that discontinuation of antihypertensive treatment in this context does not appear to be either safe or beneficial enough to be recommended in older adults with dementia.

## Supplementary Material

aa-24-0274-File004_afae133(1)

aa-24-0274-File005_afae133(1)

aa-24-0274-File006_afae133(1)

aa-24-0274-File007_afae133(1)

## Data sharing statement:

Access to the de-identified individual participant dataset and corresponding data codebook is available through the Data Archiving and Networked Services (DANS), the Dutch national centre of expertise and repository for research data. After registration on DANS, submission of a reasonable request with the provision of a methodologically sound proposal and final approval from the DANTON team, the data will be accessible on the platform. To permit the authors to perform additional analyses on the study data, an embargo period of 24 months after publication of the main trial results should elapse before one makes a request.

URL Data Archiving and Networked Services: https://easy.dans.knaw.nl/ui/advancedsearch

DOI of dataset: 10.17026/dans-xkk-zem6

URL Full protocol: https://osf.io/45eg8

URL Statistical analysis plan: https://osf.io/2gvmq
