# Supplementary material for: Effects of the discontinuation of antihypertensive treatment on neuropsychiatric symptoms and quality of life in nursing home residents with dementia (DANTON): a multicentre, open-label, blinded-outcome, randomised controlled trial
Source: Age Ageing. 2024 Jul 6;53(7):afae133. doi: 10.1093/ageing/afae133 (PMC11227112; doi:10.1093/ageing/afae133)
Supplement: aa-24-0274-File004_afae133(1) [file aa-24-0274-file004_afae133(1).docx]

**Effects of the discontinuation of antihypertensive treatment on neuropsychiatric symptoms and quality of life in nursing home residents with dementia (DANTON): a multicentre, open-label, blinded-outcome, randomised controlled trial**

**Appendices**

**APPENDIX 1: SUPPLEMENTARY METHODS AND RESULTS**

**SUPPLEMENTARY METHODS**

**Analysis**

*Per-protocol analyses*

We performed per-protocol analyses in which all participants in the intervention group without any reduction of the prescribed antihypertensive treatment between baseline and 16-week follow-up were excluded. Control participants with any change of the prescribed antihypertensive treatment (different dose or other antihypertensive drugs) between baseline and 16-week follow-up were also excluded.

*Sensitivity analyses*

As a sensitivity analysis, we repeated the analysis of the primary outcomes with adjustments for sex, history of cardiovascular disease, and for baseline variables that showed a relevant imbalance between the randomisation groups. Relevant imbalances were defined as a difference greater than the reported overall SD or greater than 10% of the reported overall median of the baseline variable. The protocol included that the missing data would be imputed. As the number of missing values in the outcomes was very limited, only complete case analyses were performed.

*Hazard ratios for cardiovascular events*

Cox proportional-hazards models were used to obtain hazard ratios (HRs) to compare the randomisation groups for the first cardiovascular event and fatal cardiovascular events. No adjustments were made in these analyses.

**SUPPLEMENTARY RESULTS**

*Per-protocol analyses*

Per-protocol analyses of the primary outcomes (see Appendix 2, Supplementary Table S7) and incidences of all-cause SAE and mortality showed results comparable to the intention-to-treat analyses (see Appendix 2, Supplementary Table S8).

*Sensitivity analyses*

In the sensitivity analyses, we repeated the primary analyses with supplementary adjustments for sex, history of cardiovascular disease, number of antihypertensive drugs, neuropsychiatric symptoms, and professional caregiver distress. As baseline differences in statin use and dementia type were above 10%, those factors were also added to the model. This analysis further confirmed the difference in change of neuropsychiatric symptoms (adjusted mean difference 5.9 [95% CI 1.3 to 10.5]; p=0.013) and the trend towards a lower quality of life in the discontinuation group at 32 weeks (adjusted mean difference -3.5 [95% CI -8.2 to 1.2]; p=0.14) (see Appendix 2, Supplementary Table S9).

*Hazard ratios for cardiovascular events*

At 16 weeks, the unadjusted HRs for the first cardiovascular event and fatal cardiovascular events were both 2.59 (95% CI 0.50 to 13.32; p=0.26). At 32 weeks, the unadjusted HRs were 1.73 (95% CI 0.58 to 5.16; p=0.32) for the first cardiovascular event and 1.30 (95% CI 0.41 to 4.09; p=0.66) for fatal cardiovascular events.

**APPENDIX 2: SUPPLEMENTARY TABLES AND FIGURES**

**Table of contents**

**Supplementary Figure S1: Semi-protocolised discontinuation protocol of antihypertensive treatment5**

Supplementary Table S1: Prescription of psychotropic drugs at baseline.6

Supplementary Table S2: Prescription details of antihypertensive treatment7

Supplementary Table S3: Prescription of psychotropic drugs at follow-up8

Supplementary Table S4: Effects of the intervention on the prescription of psychotropic drugs9

Supplementary Table S5: Details on discontinuation participants with a reported serious adverse event10

Supplementary Table S6: Details on usual care participants with a reported serious adverse event12

Supplementary Table S7: Stratified per-protocol analyses of the primary outcomes14

Supplementary Table S8: HRs for all-cause SAE and all-cause mortality of per-protocol populations…………...15

Supplementary Table S9: Sensitivity analyses of primary outcomes16

- **33 (32.7%) vs 23 (22.1%)**
- P-valure Log Rank = **0.135**
- Adjusted HR =

**
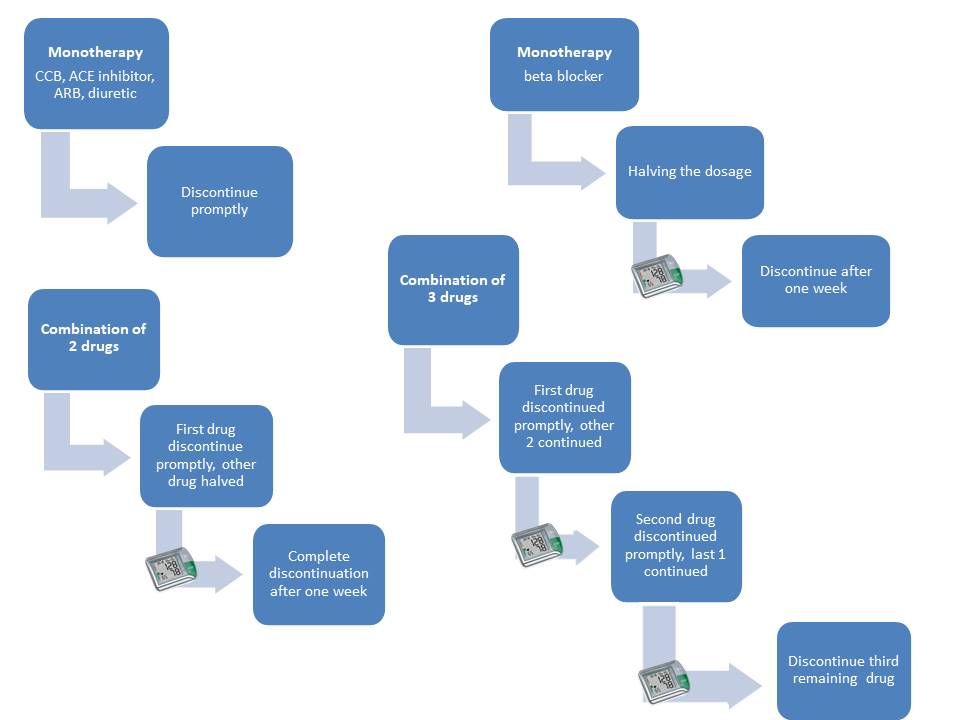
Supplementary Figure S1: Schedule of the semi-protocolised discontinuation protocol of antihypertensive treatment in the DANTON study**

ACE = Angiotensin-converting enzyme. ARB = Angiotensin receptor blocker. CCB = Calcium channel blocker

| **Supplementary Table S1: Prescription of psychotropic drugs at baseline** | | |
| --- | --- | --- |
| **Psychotropic drug** | **Discontinuation (n=101)** | **Usual Care (n=104)** |
| Antidepressants, n (%) | 32 (31.7) | 27 (26.0) |
| Antiepileptic drugs, n (%) | 5 (5.0) | 5 (4.8) |
| Antipsychotics, n (%) | 29 (28.7) | 23 (22.1) |
| Cholinesterase inhibitors & memantine, n (%) | 7 (6.9) | 2 (1.9) |
| Hypnotics & Sedatives, n (%) | 32 (31.7) | 39 (37.5) |

| **Supplementary Table S2: Prescription details of antihypertensive treatment at baseline, 16 weeks and 32 weeks of follow-up after randomisation** | | | | | | | |
| --- | --- | --- | --- | --- | --- | --- | --- |
| **Antihypertensive drug** | **Discontinuation** | | |  | **Usual Care** | | |
|  | **Baseline***  **(n=86)** | **16 weeks**  **(n=86)** | **32 weeks**  **(n=50^†^)** |  | **Baseline***  **(n=91)** | **16 weeks**  **(n=91)** | **32 weeks**  **(n=65^†^)** |
| **ACE Inhibitor, n (%)** | 36 (41.9) | 5 (5.8) | 5 (10.0) |  | 34 (37.4) | 32 (35.2) | 20 (30.8) |
| **Angiotensin receptor blocker, n (%)** | 20 (23.3) | 2 (2.3) | 1 (2.0) |  | 17 (18.7) | 16 (17.6) | 8 (12.3) |
| **Beta blocker, n (%)** | 43 (50.0) | 15 (17.4) | 7 (14.0) |  | 45 (49.5) | 41 (45.1) | 29 (44.6) |
| **Calcium channel blocker, n (%)** | 22 (25.6) | 4 (4.7) | 1 (2.0) |  | 27 (29.7) | 25 (27.5) | 16 (24.6) |
| **Loop diuretic, n (%)** | 11 (12.8) | 11 (12.8) | 7 (14.0) |  | 12 (13.2) | 14 (15.4) | 11 (16.9) |
| **Potassium sparing diuretic, n (%)** | 4 (4.7) | 1 (1.2) | 0 |  | 3 (3.3) | 2 (2.2) | 2 (3.1) |
| **Thiazide diuretic, n (%)** | 16 (18.6) | 0 | 0 |  | 14 (15.4) | 14 (14.3) | 8 (12.3) |
|  | | | | | | | |
| ACE = Angiotensin-converting enzyme. *Only baseline data of the 177 participants that completed the 16-week follow-up measurement are described. **^†^**Medication data at 32 weeks after randomisation was available for 50 out of 52 participants in the discontinuation group and 65 out of 66 in the usual care group. | | | | | | | |

| **Supplementary Table S3: Prescription details of psychotropic drug use at baseline, 16 weeks and 32 weeks of follow-up after randomisation** | | | | | | | |
| --- | --- | --- | --- | --- | --- | --- | --- |
| **Psychotropic Drug** | **Discontinuation** | | |  | **Usual Care** | | |
|  | **Baseline***  **(n=86)** | **16 weeks**  **(n=86)** | **32 weeks**  **(n=50^†^)** |  | **Baseline***  **(n=91)** | **16 weeks**  **(n=91)** | **32 weeks**  **(n=65^†^)** |
| **Antidepressants, n (%)** | 27 (31.4) | 27 (31.4) | 18 (36.0) |  | 25 (27.5) | 26 (28.6) | 20 (30.8) |
| **Antiepileptic drugs, n (%)** | 5 (5.8) | 4 (4.7) | 3 (6.0) |  | 4 (4.4) | 5 (5.5) | 4 (6.2) |
| **Antipsychotics, n (%)** | 26 (30.2) | 29 (33.7) | 15 (30.0) |  | 22 (24.2) | 21 (23.1) | 18 (27.7) |
| **Cholinesterase inhibitors & memantine, n (%)** | 7 (8.1) | 7 (8.1) | 4 (8.0) |  | 2 (2.2) | 2 (2.2) | 3 (4.6) |
| **Hypnotics & Sedatives, n (%)** | 26 (30.2) | 28 (32.6) | 19 (38.0) |  | 36 (39.6) | 38 (41.8) | 26 (40.0) |
|  |  |  |  |  |  |  |  |
| *Only baseline data of the 177 participants that completed the 16-week follow-up measurement are described. **^†^**Medication data at 32 weeks after randomisation was available for 50 out of 52 participants in the discontinuation group and 65 out of 66 in the usual care group. | | | | | | | |

| **Supplementary Table S4: Effects of discontinuing antihypertensive treatment on the prescription of psychotropic drugs** | | |
| --- | --- | --- |
| **Psychotropic Drug** | **Unadjusted OR (CI 95%)** | **Adjusted OR (CI 95%)*** |
| **16 weeks** | | |
| Antidepressants | 1.14 (0.60 to 2.18) | 0.84 (0.18 to 4.02) |
| Antiepileptic drugs | 0.84 (0.22 to 3.23) | 0.72 (0.14 to 3.62) |
| Antipsychotics | 1.70 (0.88 to 3.29) | 1.89 (0.60 to 5.95) |
| Cholinesterase inhibitors & memantine | 3.94 (0.80 to 19.54) | 1.24 (0.27 to 5.64) |
| Hypnotics & Sedatives | 0.67 (0.36 to 1.24) | 0.79 (0.27 to 2.31) |
|  |  |  |
| **32 weeks** | | |
| Antidepressants | 1.27 (0.58 to 2.77) | 1.80 (0.32 to 10.20) |
| Antiepileptic drugs | 0.97 (0.21 to 4.56) | 0.75 (0.09 to 5.93) |
| Antipsychotics | 1.12 (0.50 to 2.52) | 1.61 (0.34 to 7.56) |
| Cholinesterase inhibitors & memantine | 1.80 (0.38 to 8.42) | 0.58 (0.08 to 4.28) |
| Hypnotics & Sedatives | 0.92 (0.43 to 1.96) | 1.47 (0.40 to 5.47) |
|  | | |
| CI = confidence interval. OR = odds ratio. *Adjusted for prespecified factors: baseline value of the investigated outcome, baseline Neuropsychiatric Inventory Nursing Home score (binary, ≤12 vs >12) and long-term care organisation. | | |

| **Supplementary Table S5:**  **Detailed information about the participants of the discontinuation group with a reported serious adverse event.** | | | | | | | | | | | | | | | | |
| --- | --- | --- | --- | --- | --- | --- | --- | --- | --- | --- | --- | --- | --- | --- | --- | --- |
| **ID** | **Sex** | **Age**  **(yrs.)** | **Reisberg**  **score** | **History of** | | **Baseline** | | |  | **Follow-up 16 weeks** | | |  | **Description of the first serious adverse event** | **Days after randomisation** | **Labelled as cardiovascular?** |
|  |  |  |  | **CVD** | **DM** | **SBP** | **DBP** | **AHT (n)** |  | **SBP** | **DBP** | **AHT (n)** |  |  |  |  |
| I01 | F | 93 | 5 | Yes | No | 128 | 69 | 4 | | - | - | - | | Multi organ failure (dementia related). | 94 | Not to be determined |
| I02 | F | 82,4 | 5 | No | No | 130 | 60 | 2 | | 153 | 69 | 0 | | Dehydration (dementia related). | 234 | No |
| I03 | F | 86,1 | 5 | No | No | 129 | 74 | 2 | | - | - | - | | Pneumonia and pulmonary embolism. | 41 | No |
| I04 | F | 81,2 | 6 | Yes | Yes | 129 | 66 | 4 | | 170 | 74 | 3 | | Dehydration (dementia related). | 198 | No |
| I05 | F | 84,4 | 5 | Yes | No | 151 | 90 | 3 | | 132 | 80 | 1 | | Myocardial infarct or arrhythmia. | 179 | Yes |
| I09 | M | 70,2 | 5 | No | Yes | 118 | 66 | 2 | | - | - | - | | Multi organ failure (dementia related). | 27 | No |
| I10 | M | 84,8 | 6 | Yes | Yes | 116 | 55 | 2 | | 146 | 56 | 0 | | Cognitive/physical decline and refusal of intake/medication. | 211 | No |
| I12 | F | 86,9 | 5 | Yes | Yes | 152 | 79 | 2 | | - | - | - | | Pneumonia, dehydration, hyperglycemia and low fluid intake. | 71 | No |
| I14 | F | 68,6 | 5 | No | Yes | 125 | 55 | 1 | | 116 | 66 | 0 | | COVID-19 infection. | 194 | No |
| I15 | F | 75,4 | 5 | No | No | 158 | 89 | 2 | | 155 | 106 | 0 | | Dehydration by aspiration pneumonia (known Lewy Body dementia). | 241 | No |
| I16 | F | 89,1 | 5 | Yes | No | 126 | 72 | 1 | | 123 | 60 | 0 | | Bedridden, overall decline and severe decubitus. | 171 | No |
| I17 | F | 73,6 | 5 | No | No | 140 | 88 | 2 | | 154 | 96 | 0 | | Stroke in the left hemisphere. | 191 | Yes |
| I23 | F | 80,3 | 7 | Yes | No | 129 | 93 | 1 | | - | - | - | | Dehydration caused by pneumonia and dementia. | 80 | No |
| I25 | M | 96,7 | 6 | No | No | 144 | 71 | 1 | | - | - | - | | COVID-19 infection. | 6 | No |
| I28 | F | 91,4 | 5 | Yes | No | 156 | 61 | 2 | | - | - | - | | Ceased intake resulting in overall decline and death. | 73 | No |
| I29 | F | 90,8 | 6 | Yes | No | 127 | 58 | 1 | | 121 | 66 | 0 | | Pneumonia and deep venous thrombosis. | 134 | No |
| I30 | F | 92,4 | 6 | Yes | Yes | 92 | 50 | 1 | | - | - | - | | Acute diabetic foot disease leading to death. | 112 | Yes |
| I31 | M | 92,5 | 6 | Yes | No | 132 | 81 | 1 | | 144 | 86 | 0 | | Heart failure and dementia. | 305 | Yes |
| I32 | F | 83,8 | 6 | No | No | 151 | 94 | 2 | | 120 | 75 | 0 | | Aspiration pneumonia (opted to not start antibiotics). | 264 | No |
| I35 | F | 86,9 | 5 | No | No | 145 | 87 | 2 | | 145 | 96 | 0 | | Urinary tract infection. | 210 | No |
| I37 | F | 95,1 | 6 | Yes | No | 137 | 92 | 2 | | - | - | - | | Dehydration (dementia related). | 34 | No |
| I38 | F | 87,4 | 6 | No | No | 138 | 73 | 4 | | 177 | 103 | 1 | | Intercurrent disease and end stadium dementia. | 108 | No |
| I40 | F | 87 | 6 | Yes | No | 111 | 75 | 2 | | - | - | - | | Heart failure or infection. | 96 | Yes |
| I43 | F | 80,3 | 6 | Yes | No | 159 | 94 | 2 | | 130 | 82 | 0 | | Dementia, apraxia and dehydration. | 174 | No |
| I44 | F | 86,4 | 5 | Yes | No | 135 | 83 | 1 | | - | - | - | | Brainstem infarction. | 64 | Yes |
| I47 | F | 81,4 | 7 | No | Yes | 124 | 59 | 1 | | - | - | - | | COVID-19 infection. | 9 | No |
| I48 | F | 92,4 | 6 | Yes | No | 124 | 72 | 2 | | - | - | - | | Stroke (anticoagulation stopped due to hemorrhages). | 55 | Yes |
| I49 | F | 88,7 | 6 | No | No | 114 | 53 | 3 | | 150 | 75 | 1 | | Dehydration. | 205 | No |
| I50 | F | 86,9 | 6 | Yes | No | 133 | 77 | 1 | | 105 | 71 | 0 | | Traumatic pelvic and medial collum fracture. | 218 | No |
| I51 | F | 83,2 | 6 | No | No | 130 | 67 | 3 | | 165 | 56 | 1 | | End stadium heart failure with strong dilated aorta. | 236 | Yes |
| I52 | F | 85,5 | 5 | No | No | 153 | 94 | 2 | | - | - | - | | Arrhythmia and cardiogenic shock. | 98 | Yes |
| I56 | F | 75,6 | 6 | No | Yes | 110 | 62 | 1 | | 130 | 83 | 0 | | COVID-19 infection. | 166 | No |
| I57 | F | 78,1 | 6 | Yes | Yes | 137 | 67 | 1 | | 151 | 84 | 0 | | Traumatic medial collum fracture. | 18 | No |
| I58 | F | 79,1 | 6 | No | No | 121 | 64 | 1 | | 167 | 109 | 0 | | COVID-19 infection. | 135 | No |
| I60 | F | 72,5 | 5 | Yes | No | 152 | 82 | 1 | | 124 | 92 | 1 | | Metastasised cancer of unknown primary origin. | 216 | No |
| I61 | F | 93,9 | 6 | Yes | No | 134 | 85 | 2 | | - | - | - | | Fall. | 34 | No |
| AHT = antihypertensive treatment. CVD = cardiovascular disease. DBP = diastolic blood pressure. DM = diabetes mellitus. F = female. M = male. SBP = systolic blood pressure. Yrs = years. | | | | | | | | | | | | | | | | |

| **Supplementary Table S6:**  **Detailed information about the participants of the usual care group with a reported serious adverse event.** | | | | | | | | | | | | | | | | |
| --- | --- | --- | --- | --- | --- | --- | --- | --- | --- | --- | --- | --- | --- | --- | --- | --- |
| **ID** | **Sex** | **Age**  **(yrs.)** | **Reisberg**  **score** | **History of** | | **Baseline** | | |  | **Follow-up 16 weeks** | | |  | **Description of the first serious adverse event** | **Days after randomisation** | **Labelled as cardiovascular?** |
|  |  |  |  | **CVD** | **DM** | **SBP** | **DBP** | **AHT (n)** |  | **SBP** | **DBP** | **AHT (n)** |  |  |  |  |
| C06 | F | 86,3 | 5 | No | No | 122 | 82 | 2 | | 148 | 69 | 2 | | Not totally clear, possible cardiac cause. | 165 | Not to be determined |
| C07 | F | 88,3 | 5 | Yes | No | 119 | 45 | 2 | | 117 | 48 | 2 | | Ileus linked to intestinal tumor, deceased after palliative sedation. | 199 | No |
| C08 | F | 80,5 | 6 | Yes | Yes | 116 | 59 | 2 | | 133 | 73 | 2 | | Cardiac decompensation after hip fracture. | 213 | Yes |
| C11 | F | 92,6 | 6 | Yes | No | 134 | 70 | 2 | | - | - | - | | COVID-19 infection. | 120 | No |
| C13 | F | 87,9 | 7 | Yes | No | 126 | 46 | 1 | | - | - | - | | Dehydration and cachexia. | 56 | No |
| C18 | F | 89,7 | 6 | No | Yes | 128 | 74 | 2 | | 137 | 69 | 2 | | Dehydration and cachexia. | 231 | No |
| C19 | F | 77 | 6 | Yes | No | 147 | 75 | 2 | | - | - | - | | Dehydration after severe fall (refrained from further clinical work-up). | 96 | No |
| C20 | M | 76,8 | 7 | Yes | No | 134 | 80 | 2 | | - | - | - | | Aspiration pneumonia (long term history of swallowing difficulties). | 52 | No |
| C21 | F | 76 | 5 | Yes | Yes | 140 | 79 | 3 | | - | - | - | | COVID-19 infection. | 4 | No |
| C22 | F | 77,2 | 6 | Yes | No | 138 | 70 | 2 | | - | - | - | | Severe dementia and wounds caused by peripheral artery disease. | 63 | Yes |
| C24 | F | 95,3 | 5 | Yes | Yes | 146 | 79 | 1 | | - | - | - | | Suspicion of recurrent bowel cancer. | 32 | No |
| C26 | F | 86,8 | 5 | No | No | 146 | 86 | 1 | | - | - | - | | Renal insufficiency caused by lowered intake. | 120 | No |
| C27 | F | 97,5 | 5 | No | No | 150 | 78 | 1 | | - | - | - | | Heart failure, potentially as a result of an undiagnosed vitium cordis. | 63 | Yes |
| C33 | F | 88,6 | 6 | Yes | Yes | 141 | 73 | 3 | | - | - | - | | Overall cognitive decline and urinary tract infection. | 68 | No |
| C34 | F | 92,2 | 7 | Yes | No | 132 | 62 | 1 | | 124 | 74 | 1 | | Infection with unknown focus with hypothermia and cachexia. | 206 | No |
| C36 | F | 87,7 | 6 | No | No | 125 | 72 | 3 | | - | - | - | | Progressive decline, lowered intake and skeletal pain (metastasis). | 32 | No |
| C39 | M | 76,2 | 5 | No | No | 149 | 79 | 2 | | 190 | 75 | 2 | | Traumatic collum fracture. | 12 | No |
| C41 | M | 76,4 | 5 | Yes | No | 116 | 64 | 4 | | 86 | 56 | 4 | | Terminal delirium (probably due to metastasised cancer). | 149 | No |
| C42 | F | 76,3 | 5 | No | Yes | 135 | 87 | 2 | | - | - | - | | Long term cachexia, general decline and painful liver cysts. | 103 | No |
| C45 | F | 93,8 | 5 | Yes | Yes | 125 | 66 | 1 | | 137 | 61 | 1 | | Stroke in the right hemisphere. | 169 | Yes |
| C46 | F | 80,9 | 7 | Yes | No | 146 | 86 | 1 | | 154 | 79 | 0 | | Acute diarrhea with imminent dehydration. | 103 | No |
| C53 | F | 87,6 | 6 | Yes | No | 137 | 64 | 1 | | 145 | 102 | 1 | | Sudden unexpected death during sleep. | 233 | Yes |
| C54 | F | 77,1 | 6 | Yes | No | 117 | 74 | 1 | | 153 | 86 | 1 | | COVID-19 or ischemic stroke with secondary aspiration pneumonia. | 173 | Not to be determined |
| C55 | F | 93,4 | 6 | Yes | No | 148 | 83 | 1 | | 120 | 99 | 1 | | Ileus leading to terminal situation. | 111 | No |
| C59 | F | 89,2 | 5 | Yes | Yes | 112 | 62 | 1 | | - | - | - | | Dementia, refusal of intake and medication | 18 | No |
| AHT = antihypertensive treatment. CVD = cardiovascular disease. DBP = diastolic blood pressure. DM = diabetes mellitus. F = female. M = male. SBP = systolic blood pressure. Yrs = years. | | | | | | | | | | | | | | | | |

| **Supplementary Table S7: Per-protocol analyses of primary outcomes at 16 and 32 weeks of follow-up stratified by neuropsychiatric symptoms, quality of life and systolic blood pressure at baseline** | | | | | | |
| --- | --- | --- | --- | --- | --- | --- |
| **Mean between-group differences of change between baseline and follow-up (95% CI)** | | | | | | |
|  | |  |  | n | Adjusted* | p* |
| **Outcome NPI-NH** | |  |  |  |  |  |
|  | **16 weeks** |  |  |  |  |  |
| Overall | |  |  | 152 | 1.2 (–3.1 to 5.6) | 0.58 |
|  | |  |  |  |  |  |
| NPI-NH | | ≤12 |  | 77 | –2.2 (–6.0 to 1.6) | 0.25 |
|  | | >12 |  | 75 | 5.4 (2.5 to –13.3) | 0.18 |
|  | |  |  |  |  |  |
| Qualidem (LT) | | <70 |  | 79 | 3.2 (–4.4 to 11.0) | 0.40 |
|  | | ≥70 |  | 73 | –1.2 (–4.8 to 2.5) | 0.53 |
|  | |  |  |  |  |  |
| SBP | | <134 |  | 80 | –3.0 (–9.0 to 3.1) | 0.33 |
|  | | ≥134 |  | 72 | 6.3 (0.0 to 12.6) | 0.050 |
|  | |  |  |  |  |  |
|  | **32 weeks** |  |  |  |  |  |
| Overall | |  |  | 103 | 4.7 (0.5 to 8.9) | 0.030 |
|  | |  |  |  |  |  |
| NPI-NH | | ≤12 |  | 50 | 3.0 (–1.3 to 7.4) | 0.17 |
|  | | >12 |  | 53 | 6.3 (–1.0 to 13.6) | 0.090 |
|  | |  |  |  |  |  |
| Qualidem (LT) | | <70 |  | 55 | 7.0 (–0.1 to 14.0) | 0.054 |
|  | | ≥70 |  | 48 | 2.2 (–2.4 to 6.8) | 0.34 |
|  | |  |  |  |  |  |
| SBP | | <134 |  | 54 | 6.2 (–0.2 to 12.8) | 0.055 |
|  | | ≥134 |  | 49 | 3.1 (–2.7 to 9.0) | 0.29 |
|  | |  |  |  |  |  |
| **Outcome Qualidem (LT)** | | | | | | |
|  | **16 weeks** |  |  |  |  |  |
| Overall | |  |  | 152 | –2.1 (–5.9 to 1.6) | 0.27 |
|  | |  |  |  |  |  |
| NPI-NH | | ≤12 |  | 77 | 3.4 (–1.4 to 8.2) | 0.16 |
|  | | >12 |  | 75 | –8.0 (–13.5 to –2.5) | 0.005 |
|  | |  |  |  |  |  |
| Qualidem (LT) | | <70 |  | 79 | –4.1 (–10.4 to 2.1) | 0.19 |
|  | | ≥70 |  | 73 | 0.0 (–4.2 to 4.1) | 0.98 |
|  | |  |  |  |  |  |
| SBP | | <134 |  | 80 | 1.2 (–3.8 to 6.3) | 0.62 |
|  | | ≥134 |  | 72 | –5.7 (–11.7 to –0.3) | 0.061 |
|  | |  |  |  |  |  |
|  | **32 weeks** |  |  |  |  |  |
| Overall | |  |  | 102 | –3.8 (–8.7 to 1.0) | 0.12 |
|  | |  |  |  |  |  |
| NPI-NH | | ≤12 |  | 49 | 1.3 (–8.2 to 5.5) | 0.69 |
|  | | >12 |  | 53 | –5.8 (–12.9 to 1.3) | 0.11 |
|  | |  |  |  |  |  |
| Qualidem (LT) | | <70 |  | 54 | –2.9 (–10.4 to 4.6) | 0.44 |
|  | | ≥70 |  | 48 | –4.4 (–10.7 to 2.0) | 0.17 |
|  | |  |  |  |  |  |
| SBP | | <134 |  | 53 | –1.8 (–8.8 to 5.1) | 0.60 |
|  | | ≥134 |  | 49 | –5.9 (–13.4 to 1.5) | 0.12 |
|  | |  |  |  |  |  |
| CI = confidence interval. LT = linearly transformed. NPI-NH = Neuropsychiatric Inventory Nursing Home. SBP = systolic blood pressure. *Adjusted for prespecified factors: baseline value of the investigated outcome, baseline NPI-NH score (binary, ≤12 vs >12) and long-term care organisation. | | | | | | |

| **Supplementary Table S8: Hazard ratios for all-cause serious adverse events and all-cause mortality of Intention-to-treat and per-protocol populations** | | | | | | | | | | | |
| --- | --- | --- | --- | --- | --- | --- | --- | --- | --- | --- | --- |
| **Intention-to-treat analysis** | | | | | |  | **Per-protocol analysis** | | | | |
| n | | | Unadjusted | Adjusted* | p* |  | n | Unadjusted | Adjusted* | p* |  |
| **HR (95% CI) for all-cause SAE** | | | | | | | | | | | |
| **16 weeks** | |  |  |  |  |  |  |  |  |  | |
| 205 | | | 1.27 (0.63 to 2.57) | 1.38 (0.67 to 2.82) | 0.38 |  | — | — | — | — | |
|  | | |  |  |  |  |  |  |  |  | |
| **32 weeks** | |  |  |  |  |  |  |  |  |  | |
| 205 | | | 1.50 (0.90 to 2.52) | 1.65 (0.98 to 2.79) | 0.062 |  | 152 | 1.93 (0.90 to 4.12) | 2.17 (0.99 to 4.78) | 0.053 | |
|  | | |  |  |  |  |  |  |  |  | |
| **HR (95% CI) for all-cause mortality** | | | | | | | | | | | |
| **16 weeks** |  | |  |  |  |  |  |  |  |  | |
| 205 | | | 1.39 (0.66 to 2.95) | 1.55 (0.73 to 3.31) | 0.25 |  | — | — | — | — | |
|  | | |  |  |  |  |  |  |  |  | |
| **32 weeks** |  | |  |  |  |  |  |  |  |  | |
| 205 | | | 1.50 (0.88 to 2.57) | 1.65 (0.95 to 2.85) | 0.074 |  | 152 | 1.59 (0.73 to 3.48) | 1.72 (0.76 to 3.85) | 0.19 | |
|  | | |  |  |  |  |  |  |  |  | |
| CI = confidence interval. HR = hazard ratio. SAE = serious adverse events. *Adjusted for age, sex, history of cardiovascular diseases, baseline Neuropsychiatric Inventory Nursing Home score (binary, ≤12 vs >12) and long-term care organisation. | | | | | | | | | | | |

| **Supplementary Table S9: Sensitivity analyses of primary outcomes at 16 and 32 weeks of follow-up** | | | | |  |
| --- | --- | --- | --- | --- | --- |
| **Mean between-group difference of change between baseline and follow-up (95% CI)** | | | | |  |
|  | Unadjusted | Adjusted* | Adjusted sensitivity analysis^†^ | P value^†^ |  |
| **NPI-NH** |  |  |  |  |  |
| 16 weeks | 0.2 (–4.2 to 4.5) | 1.6 (–2.3 to 5.6) | 1.5 (–2.6 to 5.6) | 0.47 |  |
| 32 weeks | 5.3 (0.0 to 10.5) | 6.2 (1.9 to 10.6) | 5.9 (1.3 to 10.5) | 0.013 |  |
|  |  |  |  |  |  |
| **Qualidem (LT)** |  |  |  |  |  |
| 16 weeks | –1.4 (–5.2 to 2.5) | –2.5 (–6.0 to 1.0) | –1.5 (–5.1 to 2.1) | 0.40 |  |
| 32 weeks | –1.2 (–6.4 to 4.1) | –3.5 (–8.1 to 1.1) | –3.5 (–8.2 to 1.2) | 0.14 |  |
|  |  |  |  |  |  |
| CI = confidence interval. LT = linearly transformed. NPI-NH = Neuropsychiatric Inventory Nursing Home. *Adjusted for prespecified factors: baseline value of the investigated outcome, baseline NPI-NH score (binary, ≤12 vs >12) and long-term care organisation. ^†^Adjusted for baseline value of the investigated outcome, baseline NPI-NH score (binary, ≤12 vs >12), long-term care organisation, sex, history of cardiovascular diseases, number of antihypertensives, statin use, dementia type, total NPI-NH score and NPI-NH Caregiver distress. | | | | | |
