# Supplementary material for: Effects of the discontinuation of antihypertensive treatment on neuropsychiatric symptoms and quality of life in nursing home residents with dementia (DANTON): a multicentre, open-label, blinded-outcome, randomised controlled trial
Source: Age Ageing. 2024 Jul 6;53(7):afae133. doi: 10.1093/ageing/afae133 (PMC11227112; doi:10.1093/ageing/afae133)
Supplement: aa-24-0274-File005_afae133(1) [file aa-24-0274-file005_afae133(1).pdf]

1. INTRODUCTION

Name (and sponsor's ID) of trial plus Netherlands Trial Register (NTR) number
Trial name: Danton (Discontinuation of ANtihypertensive Treatment in Older people with dementia living in a Nursing home)
Number Netherlands Trial Register: will follow

Protocol name: 'Effects of stopping antihypertensive treatment on neuropsychiatric symptoms in nursing home residents with dementia'

Sponsor: Leiden University Medical Centre (CME ID: NL65719.058.18/ZonMW (Program Memorabel, dossier number 70-73305-98-1324)

Objectives of trial, including interventions being investigated
The primary objective of this study is to assess whether discontinuation of antihypertensive treatment in nursing home residents with dementia

a) reduces NPS and improves quality of life;
b) improves general daily functioning and cognitive functioning;
c) reduces psychotropic medication use, falls, care dependency and caregiver burden;
d) is safe regarding cardiovascular events.

Outline of scope of charter
This charter describes the roles and responsibilities of the Data Safety Monitoring Board of the Danton study.

2. ROLES AND RESPONSIBILITY

A broad statement of the aims of the committee
The aim of this DSMB is to ensure safety of participants of the Danton study.

Specific roles of DSMB
To regularly monitor the safety aspects of the Danton study

3. BEFORE AND EARLY IN THE TRIAL

Whether the DSMB will have input into the protocol
No.

Whether the DSMB will meet before the start of the trial
No.

Any issues specific to the disease under study
None.

Any specific regulatory issues
The Medical Ethical Committee of the LUMC has defined this trial as a non-medication trial.

Whether members of the DSMB will have a contract
No.

4. Composition

Membership and size of DSMB
The members of the DSMB are (alphabetical order):
Dr N. van Geloven, statistician, Leiden University Medical Centre, Leiden, the Netherlands
Prof.dr. W.A. van Gool, neurologist, Academic Medical Centre, Amsterdam, the Netherlands
Prof.dr. J.W. Jukema, cardiologist, Leiden University Medical Centre, Leiden, the Netherlands

The Chair, how they are chosen and the Chair's role. (likewise, if relevant, the vice-Chairman)
Prof.dr. J.W. Jukema, Leiden University Medical Centre, Leiden, the Netherlands

Responsibility of the DSMB members
The DSMB members ensure that this DSMB-charter is correct and will check the safety reports.

Responsibility of the Principal Investigator(s)
The Principal Investigators (Prof.dr. J Gussekloo, Dr RKE Poortvliet and Prof.dr W.P. Achterberg) ensure that the trial is executed according to the protocol.

Responsibility of the Principal Investigator(s)
The studyteam will be responsible for the organization of the DSMB meetings and the preparation of the materials.

5. Relationships

Relationships with Principal Investigators, other trial committees (eg Trial Steering Committee (TSC) or Executive Committee), sponsor and regulatory bodies.
The DSMB is independent of the Principal Investigators, sponsor, and regulatory bodies.

Clarification of whether the DSMB is advisory (make recommendations) or executive (make decisions)
The DSMB is advisory to the Principal Investigators.

Payment to DSMB members
None.

The need for DSMB members to disclose information about any competing interests
None.

6. ORGANIZATION OF DSMB MEETINGS

Expected frequency of DSMB meetings
A DSMB meeting will be organized after the completion of the 4-months measurement of the first 50 participants, and thereafter after every 100 participants completed 4 months. The last meeting will be after the last participants completed the 4 months meeting.

Whether meetings will be face-to-face or by teleconference
After a first face to face meeting of the DSMB, the further DSMB meetings will be by teleconference.

How DSMB meetings will be organized, especially regarding open and closed sessions, including who will be present in each session
The DSMB meetings will have an open part (members + principal investigators) and a closed part (only members of the DSMB).

7. TRIAL DOCUMENTATION AND PROCEDURES TO ENSURE CONFIDENTIALITY AND PROPER COMMUNICATION

Intended content of material to be available
The Principal Investigators will provide the DSMB with an unblinded report of
1) bloodpressure outcomes at baseline and 4 months
2) the following events during the trial: death, stroke, TIA, myocardial infarction, and hospital admission. All participating elderly care physicians are required to provide this SAE-information about trial participants to the trial personnel within seven days after the event. The trial personnel will record the information in the study database.

Will the DSMB be blinded to the treatment allocation
No.

Who will see the accumulating data and interim analysis
All members of the DSMB will see the accumulating data

To whom the DSMB will communicate the recommendations that are reached
The DSMB communicates its recommendations to the Principal Investigators.

Whether reports to the DSMB be available before the meeting or only at/during the meeting
Reports will be available before and during the meetings

8. DECISION MAKING

What decisions/recommendations will be open to the DSMB
The DSMB can make any recommendation with regard to changing or stopping the trial.

The role of formal statistical methods, specially which methods will be used and whether they

will be used as guidelines or rules
Blood pressure (Baseline and follow-up) and type of event (death, myocardial infarction, stroke, transient ischemic attack, or any non-elective hospitalization) will be given according to treatment allocation. Possible differences between treatment groups will be evaluated statistically using standard statistical techniques. Results will be used as guideline for reaching a recommendation.

How decisions or recommendations will be reached within the DSMB
The DSMB will reach their recommendations by consensus. If no consensus can be reached, they will vote, where each member has an equally weighted vote.

What is the quorum for the DSMB for decision-making
The quorum for the DSMB for decision making is two, i.e. at least two members must be present to reach a valid decision.

Can DSMB members who cannot attend the meeting input
Yes.

Whether different weight will be given to differentiate endpoints (eg safety/efficacy)
There will be no interim analysis on the endpoint(s) of the trial. Only the stated safety outcomes will be evaluated.

9. Decision making

To whom will the DSMB report their recommendations/decisions, and in what form
After each meeting the DSMB will report their recommendation to the Principal Investigators of the trial.

Whether minutes of the meeting be made and, if so, by whom and where they will be kept
Yes, the chairman will make and keep the minutes of each meeting.

What will be done if there is disagreement between the DSMB and the body to which it reports
The Principal Investigators will decide whether it follows the recommendation of the DSMB or not.

10. After the trial

Publication of results
The results of the DSMB-meetings will not be published.

The information about the DSMB that will be included in published trial reports
Information about the recommendations of the DSMB may be included in the published reports.

Whether the DSMB will have the opportunity to approve publications, especially with respect to

reporting of any DSMB recommendation regarding termination of trial
No.

Any constraints on DSMB members divulging information about their deliberations after the trial has been published
No.
