# Supplementary material for: Effects of the discontinuation of antihypertensive treatment on neuropsychiatric symptoms and quality of life in nursing home residents with dementia (DANTON): a multicentre, open-label, blinded-outcome, randomised controlled trial
Source: Age Ageing. 2024 Jul 6;53(7):afae133. doi: 10.1093/ageing/afae133 (PMC11227112; doi:10.1093/ageing/afae133)
Supplement: aa-24-0274-File006_afae133(1) [file aa-24-0274-file006_afae133(1).pdf]

RESEARCH PROTOCOL

'Effects of stopping antihypertensive treatment on neuropsychiatric symptoms in nursing home residents with dementia'

DANTON

Discontinuation of **AN**tihypertensive **T**reatment in **O**lder people with dementia living in a **N**ursing home

PROTOCOL TITLE	**'Effects of stopping antihypertensive treatment on neuropsychiatric symptoms in nursing home residents with dementia'**
**Protocol ID**	NL65719.058.18
**Short title**	DANTON
**EudraCT number**	Not applicable
**Version**	8
**Date**	26-05-2020
**Coordinating investigator/ project leader**	**Prof. dr. J. Gussekloo**<br>Director master Vitality and Ageing<br>Dept. of Public Health and Primary Care<br>Dept. of of Internal Medicine, section Gerontology and Geriatrics<br>Leiden University Medical Center<br>PO box 9600, 2300 RC Leiden<br>Tel: 071-5268444<br>E-mail: j.gussekloo@lumc.nl
**Principal investigator(s)**	**Prof. dr. J. Gussekloo**<br>Director master Vitality and Ageing<br>Dept. of Public Health and Primary Care<br>Dept. of of Internal Medicine, section Gerontology and Geriatrics<br>Leiden University Medical Center<br>PO box 9600, 2300 RC Leiden<br>Tel: 071-5268444<br>E-mail: j.gussekloo@lumc.nl<br><br>**Dr. R.K.E. Poortvliet**<br>General practitioner and senior researcher<br>Dept. of Public Health and Primary Care<br>Leiden University Medical Center<br>PO box 9600<br>2300 RC Leiden<br>Tel: 071-5268444<br>E-mail: r.k.e.poortvliet@lumc.nl

	**Prof. dr. W.P. Achterberg**<br>Dept. of Public Health and Primary Care<br>Leiden University Medical Center<br>PO box 9600<br>2300 RC Leiden<br>Tel: 071-5268444<br>E-mail: w.p.achterberg@lumc.nl
**Sponsor (in Dutch: verrichter/opdrachtgever)**	LUMC
**Subsidising party**	ZonMw Memorabel (70-73305-98-1324)
**Independent expert**	Drs. M.S. Klapwijk, elderly care physician<br>LUMC and Stichting Marente
**Laboratory sites <*if applicable*>**	Not applicable
**Pharmacy <*if applicable*>**	Not applicable
**Trial register**	This study is registered with the Netherlands Trial Registry (NTR) under number NL7365 (NTR7573)

PROTOCOL SIGNATURE SHEET

Name	Signature	Date
**Sponsor LUMC:**<br>Prof.dr J Gussekloo		04-06-2020
**Coordinating Investigator/Project leader/Principal Investigator:**<br><br>Prof. J. Gussekloo		04-06-2020
Prof. W.P. Achterberg		04-06-2020
Dr. R.K.E. Poortvliet		04-06-2020

TABLE OF CONTENTS

1. INTRODUCTION AND RATIONALE 9
2. OBJECTIVES 11
STUDY DESIGN 12
3. STUDY POPULATION 13
3.1 Population (base) 13
3.2 Inclusion criteria 13
3.3 Exclusion criteria 14
3.4 Sample size calculation 14
4. TREATMENT OF SUBJECTS 15
4.1 Intervention 15
4.2 Use of co-intervention (if applicable) 16
4.3 Escape medication (if applicable) 16
5. INVESTIGATIONAL PRODUCT 17
6. NON-INVESTIGATIONAL PRODUCT 17
7. METHODS 18
7.1 Study parameters/endpoints 18
7.1.1 Main study parameter/endpoint 18
7.1.2 Secondary study parameters/endpoints 18
7.1.3 Other study parameters (if applicable) 19
7.2 Randomisation, blinding and treatment allocation 19
7.3 Study procedures 19
7.4 Withdrawal of individual subjects 24
7.4.1 Specific criteria for withdrawal (if applicable) 24
7.5 Replacement of individual subjects after withdrawal 24
7.6 Follow-up of subjects withdrawn from treatment 24
7.7 Premature termination of the study 24
8. SAFETY REPORTING 25
8.1 Temporary halt for reasons of subject safety 25
8.2 AEs, SAEs and SUSARs 25
8.2.1 Adverse events (AEs) 25
8.2.2 Serious adverse events (SAEs) 25
8.2.3 Suspected unexpected serious adverse reactions (SUSARs) 26
8.3 Annual safety report 26
8.4 Follow-up of adverse events 26
8.5 Data Safety Monitoring Board (DSMB) 26
9. STATISTICAL ANALYSIS 28
9.1 Primary and secondary study parameter(s) 28
9.2 Other study parameters 28
9.3 Interim analysis (if applicable) 28
10. ETHICAL CONSIDERATIONS 29
10.1 Regulation statement 29

10.2 Recruitment and consent....29
10.3 Objection by minors or incapacitated subjects (if applicable)....29
10.4 Benefits and risks assessment, group relatedness....30
10.5 Compensation for injury....30
10.6 Incentives (if applicable)....31
11. ADMINISTRATIVE ASPECTS, MONITORING AND PUBLICATION....32
11.1 Handling and storage of data and documents....32
11.2 Monitoring and Quality Assurance....32
11.3 Amendments....32
11.4 Annual progress report....32
11.5 Temporary halt and (prematurely) end of study report....33
11.6 Public disclosure and publication policy....33
12. STRUCTURED RISK ANALYSIS....34
13. REFERENCES....35

SUMMARY

Rationale: Neuropsychiatric symptoms (NPS) are very common in people with dementia and severely affect quality of life and general daily functioning and hamper optimal care. They are a burden for carers and a main reason for institutionalisation. Recent studies found that hypoperfusion of the brain, hypothesised to be a result of impaired autoregulation, is related to NPS. Since antihypertensive treatment is associated with hypoperfusion of specific brain areas, increasing the blood pressure by discontinuing antihypertensive treatment is a promising treatment option for NPS, especially since 50% of the nursing home residents with dementia use antihypertensive treatment.

Objective: To assess whether discontinuation of antihypertensive treatment in nursing home residents with dementia a) reduces NPS and improves quality of life; b) improves general daily functioning and cognitive functioning; c) reduces psychotropic medication use, falls, care dependency and caregiver burden; and d) is safe regarding cardiovascular events.

Study design: Randomized non-blinded controlled clinical trial.

Study population/eligibility criteria: Residents from nursing homes can participate if they (1) have a diagnosis of moderate-severe dementia, (2) are on antihypertensive treatment, and (3) have a systolic blood pressure <=160mmHg. Older adults will be excluded if they have heart failure NYHA class III or IV, recent (<12 months) history of myocardial infarction, stroke, coronary reperfusion procedures (CABG/PCI), or have a life-expectancy less than 4 months.

Intervention: Randomization to discontinuation (n=246) or continuation (n=246) of antihypertensive treatment during 8 months. Discontinuation of antihypertensive treatment aims to achieve a systolic blood pressure increase of 20 mmHg using a drug-specific discontinuation algorithm.

Main study parameters/endpoints: The co-primary outcome measures are the differences in change of scores between 0 and 4 months on the Neuropsychiatric Inventory – Nursing Homes (NPI-NH) and quality of life. Secondary outcome measures include NPS registered in the medical records, apathy, care dependency, cognitive function, general daily functioning, care-related quality of life, orthostatic hypotension, incident falls, and psychotropic medication use. Long-term effects on primary and secondary outcomes will be analysed over 8 months. In addition, cost-effectiveness will be evaluated.

Nature and extent of the burden and risks associated with participation, benefit and group relatedness: Assessments of NPS with the NPI-NH, quality of life, dementia severity, cognitive functioning, care dependency and general daily functioning will be done at the nursing home both at baseline and at 4 and 8 months. Most questionnaires will be filled out by professional and informal caregivers of the patients to get information by proxy.

Patients in both study arms will continue to receive blood pressure measurements during follow-up after a stable blood pressure has been reached. This will be done for safety reasons in the intervention arm and to make both study arms as similar as possible also in the control arm. Patients in the intervention arm will be put on their original antihypertensive medication when diastolic blood pressure exceeds 120 mmHg or systolic blood pressure exceeds 200 mmHg (180 mmHg for participants with diabetes mellitus or those who had had a cardiovascular event >12 months ago) or an increase in systolic blood pressure of 60 mmHg or greater relative to baseline. Moreover, all cardiovascular events during the study will be closely monitored to prevent an increase in cardiovascular events in the intervention group. A Data Safety and Monitoring Board (DSMB) will be installed for monitoring of the safety data (cardiovascular events).

This study will not interfere with standard care, diagnostics and treatment (other than antihypertensive treatment) for patients with dementia.

Given the future rise in the number of older people with dementia and NPS in our society, the impact of this trial will be substantial when this trial demonstrates that NPS can be alleviated and quality of life can be improved by discontinuation of antihypertensive treatment. Since NPS hamper optimal care and are a serious burden for caregivers, this study will not only have an impact on dementia patients, but also on caregivers and nursing staff.

1. INTRODUCTION AND RATIONALE

Problem definition

There are an estimated 35 million people with dementia across the world. Currently 5% of people over 65 have a diagnosis of dementia, rising to over 50% in the 90+ group. The number of older persons in our society has risen enormously and their numbers will continue to rise (Oeppen et al., 2002). With increasing age, the prevalence of dementia increases exponentially (Ott et all., 1998). Neuropsychiatric symptoms (NPS), such as apathy, delusions, hallucinations, agitation and aggressive behaviour are highly prevalent in older persons with dementia (Ballard et al., 2001). Almost all (97%) nursing home residents with dementia have at least one NPS in a two-year period and 74% in a one-year period (Wetzels et al., 2010).Together with physical dysfunction, NPS have the highest impact on quality of life of patients with dementia and are the main reason for institutionalization. Especially in the more advanced stages of dementia, NPS become prominent, burdensome, and extremely distressing for patients, family and caregivers. Current treatment includes psychotropic medication and psychosocial interventions, with disappointing success rates (Briesacher et al., 2005). While psychotropic medication has its place in the treatment of severe or persistent NPS, its use has also been associated with substantial side effects including falls, cerebrovascular events, and death. In a review of 11 studies, the percentage of patients that experienced some sort of adverse event ranged from 49% to 100% (van Iersel et al., 2005).

The aetiology of NPS in dementia is multifactorial and includes neuropathological changes in the brain as well as unmet physical and psychological needs related to dementia (Steinberg et al., 2006). One of the causal mechanisms with potential therapeutic opportunities is the observed relationship between hypoperfusion of (specific areas of) the brain and NPS. Cerebrovascular autoregulation protects cerebral blood vessels from the wide swings in arterial pressure and provides a stable cerebral blood flow, and thus protects the brain from hypoperfusion. Hypertension and stroke can cause disturbances in cerebrovascular autoregulation due to e.g. endothelial and vascular damage (Iadecola, 2013). When autoregulation is impaired, the susceptibility of the white matter to damage during fluctuation in blood pressure increases (Matsushita et al., 1994) and hypoperfusion of (specific) brain areas occurs. The stable plateau of cerebral blood flow is narrower than originally thought (Willie et al., 2014). The cerebral blood flow reduction is observed prior to the onset of dementia (Ruitenberg et al., 2005).There is increasing evidence that hypoperfusion of the brain also plays a role in the development of NPS like apathy (Benoit et al., 1999; Craig et al., 1996; Marshall et al., 2007), depressive symptoms (Hirono et al., 1998), psychotic symptoms (Mega et al., 2000) and aggressive behaviour (Hirono et al, 2000). A high blood pressure in old age may be required to maintain adequate cerebral perfusion. Hence, blood pressure reduction in older people may lead to hypoperfusion, especially in patients with cerebral small vessel disease, resulting in increased mental health problems.

In dementia patients, the use of antihypertensive medication is associated with increased occurrence of NPS (Steinberg et al., 2014; Bassiony et al., 2000). Since a large proportion of persons with dementia have widespread cerebrovascular damage and consequently, an impaired cerebral autoregulation, the question raises about the desirability of antihypertensive treatment use in nursing home residents, which is currently up to 50% (Koopmans et al., 2003; van Dijk et al., 2000). Therefore, increasing blood pressure by discontinuing antihypertensive treatment could be a promising treatment and prevention option. Currently, there are no guidelines for the discontinuation of antihypertensive medication in patients with dementia, despite the lack of firm evidence for benefits or harm of its continued use. Available trials on (discontinuation of) antihypertensive treatment in older persons, including HYVET (Becket et al, 2008), SPRINT (Williamson et al, 2016) and DANTE (Moonen et al, 2015), excluded nursing home residents with dementia. There is an urgent need for trials to assess the benefit to risk ratio of antihypertensive treatment in the growing nursing home population (Benetos et al., 2015). The latest 2014 US (James et al., 2014) recommend less aggressive targeting of blood pressure thresholds for older, but lacks recommendations for persons aged >80 years with dementia. The European Society of Hypertension (Benetos et al., 2016) addresses the potential adverse impact of excessive blood pressure lowering in nursing home residents. The lack of evidence leads to the clinical dilemma whether or not to start, stop or continue antihypertensive treatment in older people with dementia (Muller et al, 2014).

DANTE, a recently completed study, including community-dwelling persons aged 75 years and over with mild cognitive deficits (MMSE 21-27), showed that temporary discontinuation of antihypertensive medication during 16-weeks did not result in adverse (cardiovascular) events or more hospitalisations. It showed no effect on cognitive, psychological of general daily functioning (Moonen et al., 2015). The DANTE trial included persons without dementia, without serious cardiovascular disease, and with a higher functional status; i.e. non-frail participants. Moreover, the prevalence of NPS in the DANTE trial was extremely low, wherefore power was insufficient to assess the effect of discontinuation of antihypertensive treatment on NPS. This study will be the first to assess this effect. Nursing home residents are a study population of interest because they have more advanced dementia resulting in more NPS, and have a higher prevalence of cerebrovascular disease (Benetos et al., 2015), wherefore they are prone to have a failing cerebral autoregulation. In this study, we will assess whether a 8-month discontinuation of antihypertensive medication in nursing home residents with moderate to severe dementia on antihypertensive treatment improves psychological functioning and quality of life and if it is cost-effective.

2. OBJECTIVES

NPS in dementia patients is highly prevalent, burdensome, and extremely distressing for patients, family and caregivers. There is increasing evidence that hypoperfusion of (specific areas of) the brain could be underlying NPS. Since more than half of the dementia patients do get antihypertensive treatment which has an effect on the perfusion of the brain, we question whether discontinuation of antihypertensive treatment could diminish NPS in dementia patients.

Therefore the overarching aim of the present project is to study the effects of discontinuation of antihypertensive medication in older dementia patients. We hypothesize that increasing blood pressure by discontinuation of antihypertensive treatment would reduce NPS and improves quality of life in nursing home residents with moderate to severe dementia

The co-primary outcomes of this study are neuropsychiatric symptoms in various domains measured with the Neuropsychiatric Inventory–Nursing Homes (NPI-NH) and quality of life measured with Qualidem.

The secondary outcomes are:
- NPS registered in the medical nursing home records during the study period, concomitant psychotropic medication use, psychosocial interventions started for NPS
- apathy
- general daily functioning, care dependency
- cognitive function
- orthostatic hypotension
- number of falls
- caregiver burden (formal and informal caregivers)

For the main analysis, the endpoints over four months will be used. Long-term effects on primary and secondary outcomes will be analysed over 8 months.

Safety is expressed in the number of cardiovascular events (serious adverse outcomes).
The cost-effectiveness of the intervention will be assessed from societal perspective.

By add-on qualitative studies, we will explore which factors play a role in the decision to start or stop cardiovascular preventive medication for doctors, formal care givers, informal carers and family.

STUDY DESIGN

This study is a randomized non-blinded controlled clinical trial.

Figure: Flow chart of the Danton study design

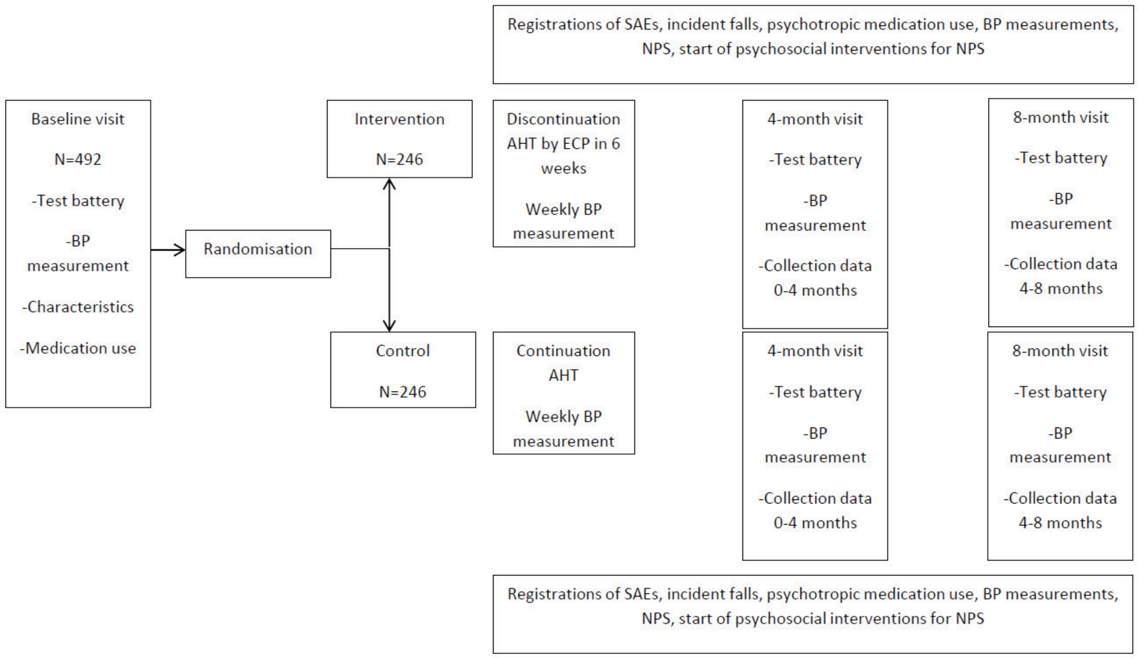

3. STUDY POPULATION

3.1 Population (base)

Our study will be conducted in 492 nursing home residents with dementia, recruited from nursing homes mainly affiliated with the University Network for the Care Sector South Holland (UNC-ZH), a university network of 10 large organisations for long-term care in the province South Holland. In total these organisations have around 50 nursing homes. This project will be part of the ongoing research activities within the UNC-ZH, and fits well within their research theme quality of life in dementia. Participants will be recruited from nursing homes from these ten long-term care organisations participating in the UNC-ZH.

Besides, the UNC-ZH is part of a national partnership called SANO. This is a national partnership between the six Academic Nursing Home Networks in the Netherlands. Each of these six Academic Nursing home Networks forms a partnership between a university (hospital) and healthcare organizations in the region. Therefore, we are able to recruit participants in the other SANO regions as well, if necessary. Participants can also be recruited from nursing homes not (yet) affiliated to university networks. All participating long-term care organisations will sign a ‘lokale uitvoerbaarheidsverklaring’.
Over a two year period, each of the long-term care organisations has around 600 residents with dementia. 80% of these patients will not fulfil the inclusion criteria since 50% will not use antihypertensive treatment and another 30% will be excluded due to other criteria (including recent cardiovascular event, heart failure class III or IV and limited life expectancy). This will result in 120 eligible participants per long-term care facility, Taking into account a refusal and drop-out rate of 60% eligible participants, this means per health care organisation 48 participants. In total, we will need 10-11 long-term care organisations. Therefore, we are confident that we will be able to recruit 492 participants over 2 years.

Diversity
Nursing home residents of all ethnic backgrounds and socio-economical classes can be included. Ethnic background and socio-economic status will be asked at baseline. Participants will be included from different nursing homes. The percentage non-Western ethnicity is high in some of these nursing homes, especially in those in The Hague and Rotterdam.

3.2 Inclusion criteria

In order to be eligible to participate in this study, a subject must meet all of the following criteria:
• have a diagnosis of moderate to severe dementia according to the Reisberg Global Deterioration Scale (score 5-6-7)
• are currently on antihypertensive treatment with a calcium antagonist, diuretic, ACE-inhibitor, beta-blocker or angiotensin-II-receptor blocker prescribed for hypertension
• have a systolic blood pressure <=160mmHg (average of two last blood pressure measurements)

3.3 Exclusion criteria

A potential subject who meets any of the following criteria will be excluded from participation in this study:

- recent (<12 months) history of myocardial infarction, stroke, coronary reperfusion procedures (CABG/PCI)
- heart failure NYHA class III or IV
- current angina pectoris
- have a life-expectancy less than 4 months.

3.4 Sample size calculation

Sample size calculation is based on the main outcome measures, the change in NPS and quality of life between baseline and follow-up at 4 months. With an alpha of 0.05, a power of 0.90, and a clinically relevant difference of 4 points (standard deviation [SD] 11) on the NPI-NH between the two groups and a clinically relevant change of 10% on the short version of the Qualidem (median 70, SD 13), a total number of 160 participants are needed in each group.

Accounting for 35% loss to follow up within four months (withdrawal or mortality), a total number of 246 participants will be included in each arm of the trial.

4. TREATMENT OF SUBJECTS

4.1 Intervention

In participants randomized to the intervention group, their treating elderly care physician will actively withdraw antihypertensive treatment. The clinical responsibility for the maximum increase in blood pressure of 20 mmHg by (partial) discontinuation of antihypertensive medications will be taken by the treating elderly care physician of the individual participant.

Antihypertensive medication discontinuation algorithm

Participants will be randomised to discontinuation or continuation of antihypertensive treatment during 8 months. Discontinuation of antihypertensive treatment in participants in the intervention arm may vary from abrupt and complete discontinuation to gradual and partial discontinuation, depending on the various categories of drugs and the absolute systolic blood pressure achieved. An increase of 20 mmHg in systolic blood pressure is the target and 180 mmHg as maximum systolic blood pressure.

For the various antihypertensive drugs commonly used by older people a discontinuation algorithm have been developed by an expert team (Moonen et al., 2015). This is presented in appendix 1. Monotherapy with a calcium channel blocker, angiotensin converting enzyme inhibitor, angiotensin receptor blocker, or diuretic will be discontinued promptly, while a beta blocker will be first halved and then stopped the next week. In the case of combination therapy of 2 drugs, first 1 drug will be discontinued promptly and the other drug will be halved, followed by complete discontinuation the next week. In the case of combination therapy of 3 drugs, first 2 drugs will be discontinued promptly and the third will be fully continued. In the consecutive 2 weeks this remaining third drug will be first halved and then completely discontinued. Discontinuation will be executed and completed within six weeks from randomization by subjects' own elderly care physician.

Discontinuation logs of the previous DANTE trial showed that a discontinuation period of six weeks is needed to reach a target of +20mmHg in systolic blood pressure by partial/complete discontinuation of antihypertensive treatment step by step in patients using >1 antihypertensive drugs.

The treating elderly care physician or nursing staff will weekly monitor the blood pressure of all participants. An expert team, including an internist of the LUMC-department Internal Medicine, section of Gerontology and Geriatrics, will advise elderly care physicians.

Training

Elderly care physicians of the nursing homes in participating long-term care organisations will receive a 2 hours training program, consisting of a general introduction about antihypertensive treatment in older patients with dementia and NPS. Communication on medication withdrawal and the discontinuation algorithm will be discussed. Furthermore, in small groups different patient cases will be discussed and experiences and opinions of elderly care physicians related to these cases will be explored. The training will provide

guidance on risk and withdrawal communication, in order to make the elderly care physician feel comfortable withdrawing antihypertensive treatment in the for the study selected patients. The interval between training and start of the intervention (withdrawal of antihypertensive treatment) will be kept as short as possible, therefore, we believe the training will have a positive effect on the elderly care physician's communication on withdrawal and their confidence in their treatment. The training will be developed by internal medical specialists of the LUMC-department Internal Medicine, section of Gerontology and Geriatrics, elderly care physicians and general practitioners. These developers will present the training, including the PhD student appointed to this project.

Qualitative study

During the development and evaluation of the training and the study as a whole, professional care givers (including nurses and elderly care physicians) and informal care givers and legal representatives of nursing home residents with dementia will be interviewed to explore the weighing of the benefit (improvement of neuropsychiatric symptoms/quality of life) versus the risk/harm (cardiovascular events) and which factors play a role in the decision to start or stop cardiovascular preventive medication. These interviews will be semi-structured. If better applicable, the qualitative data will be gathered by conducting focus groups. We will continue with this data collection until data-saturation is achieved. The researchers will explain participants of the interviews or focus groups (professional caregivers and legal representatives of nursing home residents with dementia) that all that is said during the procedure is confidential and that after analysis the audio-tapes will be destroyed. The data will be analysed with help of ATLAS.ti. Data will be coded by two researchers. Inequalities in coding will be solved by discussion.

4.2 Use of co-intervention (if applicable)

Participants in the intervention group are allowed to use co-medication other than antihypertensive treatment. Co-interventions to reduce NPS, like psychosocial interventions can be continued or started, this will be registered and is a secondary outcome. The use of concomitant psychotropic medication is also allowed and will be registered as a secondary outcome. Participants in the intervention group will be put on their original antihypertensive medication when diastolic blood pressure exceeds 120 mmHg or greater, or systolic blood pressure exceeds 200 mmHg or greater (180 mmHg for participants with diabetes mellitus or those who had had a cardiovascular event >12 months ago), or an increase in systolic blood pressure of 60 mmHg or greater relative to baseline. Intention to treat analysis will be performed.

4.3 Escape medication (if applicable)

Not applicable.

5. INVESTIGATIONAL PRODUCT

Not applicable

6. NON-INVESTIGATIONAL PRODUCT

Not applicable

7. METHODS

7.1 Study parameters/endpoints

The endpoint concerning the patients will be assessed by measurements of the patients and information from professional and informal caregivers by (blinded) research personnel at baseline and 4 and 8 months, from medical records and from pharmacy files. For an overview see appendix 2

7.1.1 Main study parameter/endpoint

The co-primary outcomes of this study are neuropsychiatric symptoms (NPS) and quality of life. NPS will be assessed with the NPI-NH (Kat et al., 2002; Cummings, 1994; Cummings, 1997)) by (blinded) research personnel. Quality of life will be assessed with the Qualidem (Ettema et al., 2007). The difference in NPI-NH and quality of life at 4 months between intervention and control is the co-primary outcome measure. The outcome measurements at 0, 4 and 8 months will be assessed by blinded research personnel.

7.1.2 Secondary study parameters/endpoints

General daily functioning will be assessed with the Katz Index of Independence in activities of daily living (Katz et al 1970); Care dependency will be assessed with the Care Dependency Scale (Dijkstra et al., 1999). Cognitive functioning will be measured by the 7-category Minimum Data Set Cognitive Performance Scale (Morris et al., 1994) and presence of delirium signs will be assessed with the NH-CAM (Dosa et al., 2007). Apathy will be measured with the abbreviated Apathy Evaluation Scale (Lueken et al,, 2007). The EQ-5D+C will be used to measure quality of life (Krabbe et al 1999), and is necessary for the cost-evaluation.

In principle, the blood pressure will be measured at baseline, 4 and 8 months by research personnel. In circumstances when the nursing home is not accessible for external visitors (e.g. when a pandemic prevention plan is activated), the blood pressure measurements at baseline and 4 and 8 months after randomisation will be carried out by the elderly care physician or a professional caregiver from the nursing home. Blood pressure will be measured standardised twice in sitting position using a digital sphygmomanometer on the right arm (except when there is a contraindication to measure it on the right arm). If possible, blood pressure will also be measured standing (three times) to assess orthostatic hypotension. Orthostatic hypotension will be defined as a drop of at least 20 mmHg in systolic blood pressure and/or 10 mmHg in diastolic blood pressure on standing from a seated position.

Care-related quality of life and caregiver burden will be assessed with the CarerQoL-7D. (Brouwer et al, 2006; Hoefman et al 2011)

In addition, nursing staff and elderly care physicians will be asked to record NPS during the study period as registered in the medical records. Concomitant

psychotropic medication use and the start of psychosocial interventions for NPS will also be assessed from the medical records and pharmacy files. Furthermore, incident falls will be gathered from the medical records and nurse records to compare the incidence of falling between the intervention and the control arm.

7.1.3 Other study parameters (if applicable)

Demographic and clinical characteristics will be collected at baseline, including age; sex; ethnicity; socio-economic status; smoking history; history of vascular disease; somatic co-morbidity; total medication use (besides psychotropic medication).

7.2 Randomisation, blinding and treatment allocation

All participants will be randomized on a 1:1 ratio to parallel discontinuation (intervention group n=246) or continuation (control group n=246) of antihypertensive treatment.

Stratified block randomization will be used (with variable block sizes per nursing home) to ensure that intervention and control participants were equally distributed within nursing homes. Additional stratified block randomization will be used (with variable block sizes) to ensure that intervention and control patients were equally distributed according to their severity of NPS at baseline.

Concealment of treatment allocation will be ensured by a central computerized randomization procedure. Participants and caregivers will not be blinded to treatment allocation.

Research personnel will be blinded for treatment allocation. Discontinuation of antihypertensive treatment will be carried out by participants' treating elderly care physician. This will minimize the possibility that information bias will affect the assessment of outcome measures.

7.3 Study procedures

Participant enrolment

Subjects will be recruited from nursing homes of the UNC-ZH. We will follow the procedures as been have developed and approved by the UNC-ZH and CME for protocol P17-051.

An in- and exclusion list will be provided to the treating elderly care physician. This list will help the treating elderly care physician in the selection of potentially eligible residents. The list contains all in- and exclusion criteria; gender (male/female); age in 4 categories (<80; 80-84; 85-89 and ≥90); and 4 categories of cardiovascular preventive medication (including the inclusion criteria antihypertensive treatment). The

completed lists will be collected by the research team after anonymization in order to be able to complete the flowchart for in- and exclusion of the trial.
Eligible residents and their legal representatives will be selected by the treating elderly care physician, after which the legal representative receives the Patient Information letter, that explains the purpose and procedures of the proposed study, the tests and questionnaires required and possible hazards that may be involved.

The legal representative will be asked to return the consent form to the researchers by mail, either with a consent, or with refusal for the patient to participate. If the legal representative sends his/her consent, the researcher or research nurse will contact the legal representative by telephone to assure the right person has signed the form and to answer questions if necessary. If the researcher or research nurse has assured him/herself, the researcher or research nurse will sign the consent form as well. A copy will be retained by each legal representative and added to the medical record of the patient in the nursing home. Thereafter the resident will be enrolled in the study. Of all subjects enrolled in the study, a case report form is kept.

Intervention
The elderly care physician will be informed by one of the members of the research centre, who is unblinded, about the outcome of randomisation. Within six weeks from randomisation discontinuation will be executed and completed by patients' own elderly care physician, strictly according to discontinuation protocol. During the discontinuation period of six weeks blood pressure will be monitored weekly until a stable blood pressure has been reached. Thereafter, participants in the discontinuation arm will continue to receive regular blood pressure measurements according to protocol for safety monitoring. Participants in the continuation arm will also receive regular blood pressure measurements.

Measurements
After informed consent, baseline information, blood pressure and baseline measurements will be gathered by research nurses. Thereafter, patients will be randomized to either continuation or discontinuation of antihypertensive treatment. At four and eight months after randomisation, all participants will be visited again by the research nurse for assessment of all outcome measures.

Most data will be collected from formal and informal caregivers. In Dutch nursing homes, each nursing home resident is appointed to a so-called 'first responsible nurse', this nurse is coordinating the care for this resident. This nurse will be involved in the assessments. In addition, the research nurses appointed to this project will help to coordinate and will take (part off) the assessments. In circumstances when the nursing home is not accessible for external visitors (e.g. when a pandemic prevention plan is activated), the research nurse will contact the 'first responsible nurse' for the assessment of all outcome measures during a telephone interview. In such set of

conditions, the blood pressure measurements at baseline and 4 and 8 months after randomisation will be carried out by the elderly care physician or the ‘first responsible nurse’.

Instruments

Neuropsychiatric Inventory–Nursing Homes (Cummings, 1994)

The Neuropsychiatric Inventory–Nursing_Homes (NPI-NH) is based on a structured interview with an informant (a nurse in our study) and assesses presence and severity of 12 NPS domains: Delusions; Disinhibition; Hallucinations; Irritability/ Lability; Agitation/Aggression; Aberrant motor behaviour; Elation/Euphoria; Apathy/Indifference; Depression/Dysphoria; Anxiety; Sleep and Night-time Behaviour Disorders; Appetite and Eating Disorders.

For each domain there are four scores: frequency, severity, total (frequency x severity, with a maximum score of 12 points per item, and 144 in total). The Dutch version of the NPI has high interrater agreement and is found to be a valid rating scale for measuring a wide range of behavioural and psychological symptoms of dementia. Next to the frequency and severity scores, we will also use the Caregiver Distress Scale of the NPI, which assesses the level of caregiver (occupational) distress associated with the subjects’ behavioural disturbances measured with the NPI, ranging from 0 (no distress) to 60 (very disruptive, major source of distress for staff) (Zuidema et al, 2007).

Qualidem (Ettema et al., 2007)

Qualidem is a quality of life measure for people with dementia within residential settings rated by professional caregivers. The original Qualidem consists of 37 items describing observable behaviour. In this study we will use the 18 items that are also applicable for very severe dementia (GDS 7), as the authors did recommend in the manual of the QUALIDEM. These 18 items cover 6 QoL domains (care relationship, positive affect, negative affect, restless tense behaviour, social relations, and social isolation). The QUALIDEM (18 and 37 items) is one of the few QoL instruments that focuses on the QoL domains that are judged important for PWD, even in severe end-stage dementia, and therefore is a suitable instrument for the evaluation of QoL in PWD (Dichter et al, 2014).

DS-DAT (Hurley et al 1992)

As recommended, we use the QUALIDEM together with the DS-DAT (a measure to assess discomfort in dementia) to evaluate the influence of interventions and 24-h care on QoL in severe dementia (ref). The DS-DAT is a 9-item observational instrument that measures discomfort symptoms of patients, regarding vocalizations, breathing, facial expression, and body movement. The Dutch version appeared to be suitable assessing discomfort in nursing home residents that have severe dementia, and it has proven to be valid and reliable (Hurley et al 1992; van der Steen et al 2002).

EuroQol-5D+C (EuroQol Group, 1990, Krabbe et al 1999)

For the purposes of this project, a modified version of the EQ-5D, the EQ-5D+C will be used (Krabbe et al 1999). Whereas the traditional EQ-5D assesses five attributes (mobility, selfcare, usual activities, pain/discomfort, anxiety/depression), the EQ-5D+C includes an additional attribute to assess cognitive function. The EQ-5D+C generic health index comprises a six-part questionnaire and a visual analogue self-rating scale. The questionnaire may be used as a health index to calculate a 'utility' value or as a health profile. It is used in economic evaluations. In this study a formal caregiver as a proxy will be asked to indicate the level of health by checking one of three boxes for each domain. For the VAS, informal caregivers draw a line from a box to the point on the thermometer like-scale corresponding to their health stat, 0-100 (100 is best health state) (Oostenbrink 2004). It has been used successfully in a large nursing home population in the UNC-ZH network (Caljouw et al, 2014)

7-category Minimum Data Set Cognitive Performance Scale (CPS) (Morris et al., 1994)

The CPS scale combines selected MDS cognitive items within a hierarchical 7-category rating scale, ranging from no cognitive impairment to very severe impairment. Modelling of the CPS was based on two standard cognitive assessment instruments; the Mini-Mental State Examination and the Test for Severe Impairment. The MDS Cognitive Performance Scale provides a valid proxy rated observational measure of cognitive status in nursing home residents (Hartmaier et al., 1995).

Nursing Home Confusion Assessment Method (Dosa et al., 2007)

The Nursing Home Confusion Assessment Method (NH-CAM) was developed for diagnosing delirium using items found on the Minimum Data Set. It is designed to allow non-psychiatric clinicians to diagnose delirium quickly and accurately following brief formal cognitive testing. The NH-CAM instrument assesses the presence, severity, and fluctuation of 9 delirium features: acute onset, inattention, disorganized thinking, altered level of consciousness, disorientation, memory impairment, perceptual disturbances, psychomotor agitation or retardation, and altered sleep-wake cycle.

Care Dependency Scale (Dijkstra et al., 1999, 2000, 2002, 2005)

The Care Dependency Scale (CDS) is a tool completed by nursing staff for assessment of the care dependency status of institutionalized residents [31]. Content of the CDS consists of 15 items: Eating and drinking; Avoidance of danger; Continence; Communications; Body posture; Contact with others; Mobility; Sense of rules and values; Day/night pattern; Daily activities; Getting dressed and undressed; Recreational activities; Body temperature; Learning activities; Hygiene. All categories are marked using a 5-point Likert-type scale. Responses range from being ‘1 = completely dependent’ to ‘5 = almost independent’. The total CDS score ranges from 15 (completely dependent on care) to 75 (almost independent of care). CDS has satisfactory reliability and validity.

Katz Index of Independence in activities of daily living (Katz et al.1963)

The Katz ADL index is an instrument to assess functional status as a measurement of the participants ability to perform activities of daily living independently. The Index ranks adequacy of performance in the six functions of bathing, dressing, toileting, transferring, continence, and feeding. The functions can be scored yes/no for independence. A score of 6 indicates full function, 4 indicates moderate impairment, and 2 or less indicates severe functional impairment. The summary score of the Katz ADL index ranges from 0 (low function/fully dependent) to 15 (high function/fully independent). It is a reliable and valid instrument for measuring ADL-function (Katz et al 1963). It has also been shown to be both reliable and sensitive to change in persons with dementia (Katz et al, 1970; (Laan et al, 2014). The questionnaire is filled out by nursing staff.

Care-related Quality of Life instrument (Brouwer et al., 2006)

The CarerQol-7D instrument produces care-related quality-of-life scores of caregivers which take differences in the importance of problems that caregivers can face into account. The CarerQol-7D measures two positive dimensions of caregiving (fulfilment and support) and five problem dimensions (relational problems, mental health, physical health, financial problems, and problems combining daily activities with caring), each with three response categories: no; some; a lot.

Reisberg Global Deterioration Scale (Reisberg et al, 1982)

This scale is developed for the assessment of primary degenerative dementia and delineation of its stages. A score of 5 indicate moderate dementia; a score of 6 indicate moderately severe dementia and a score of 7 indicate severe dementia. The Reisberg GDS scale will be used to assess the severity of dementia. To be eligible to participate in this study, a subject must have a diagnosis of moderate to severe dementia according to the Reisberg Global Deterioration Scale (score 5, 6 or 7).

The Abbreviated Apathy Evaluation Scale-10 (Lueken et al,, 2007)

The abbreviated Apathy Evaluation Scale-10 (AES-10)_consists of 10 observational items describing apathetic behaviour. Each behavioural item can be scored from 1 (not at all characteristic) to 4 (a lot characteristic). A total score ranges from 10 to 40, with a higher score reflecting a higher presence of apathy. This scale has been validated in a Dutch nursing home population with dementia (Leontjevas et al, 2012).

Information on medical history, medical events and medication

For this study, information on medical history, medical events and use of medication will be gathered from the medical records in the nursing home by the elderly care physician and/or research nurse.

End of the intervention

After a follow-up period of 8 months, the elderly care physician decides in agreement with the legal representative wether or not the participants in the discontinuation arm will restart their antihypertensive treatment. Reintroduction of antihypertensive treatment will be performed to the insights of the treating elderly care physician.

To evaluate the post-trial decision-making on antihypertensive treatment, additional data (registered in the medical nursing home files) on medication use will be collected in all participants until 4 months after the end of the intervention. In addition, cardiovascular events and (when possible causespecific) mortality will also be monitored after the end of the intervention.

7.4 Withdrawal of individual subjects

Subjects can leave the study at any time for any reason if they wish to do so without any consequences of their daily care. The investigator can decide to withdraw a subject from the study for urgent medical reasons.

7.4.1 Specific criteria for withdrawal (if applicable)

Not applicable

7.5 Replacement of individual subjects after withdrawal

Newly recruited subjects will replace withdrawals in case the withdrawal is before the randomisation.

7.6 Follow-up of subjects withdrawn from treatment

If a subject withdraws, the research nurses will record the withdrawal on the case report form. Measurements will be continued if possible and if accepted by the participant and/or its relative, following the intention to treat principle.

7.7 Premature termination of the study

The DSMB will decide, together with the principal investigator, whether there is a safety reason for premature termination.

8. SAFETY REPORTING

8.1 Temporary halt for reasons of subject safety

In accordance to section 10, subsection 4, of the WMO, the sponsor will suspend the study if there is sufficient ground that continuation of the study will jeopardise subject health or safety. The sponsor will notify the accredited METC without undue delay of a temporary halt including the reason for such an action. The study will be suspended pending a further positive decision by the accredited METC. The investigator will take care that all subjects are kept informed.

8.2 AEs, SAEs and SUSARs

8.2.1 Adverse events (AEs)

Adverse events are defined as any undesirable experience occurring to a subject during the study, whether or not considered related to discontinuation of antihypertensive treatment. All adverse events reported spontaneously by the subject or observed by the investigator, his staff or the elderly care physician will be recorded on the AE data collection form.

At each study visit, the Investigator/research personnel registers possible AEs and side effects of discontinuation of antihypertensive treatment by structured questionnaires. The research personnel will consult the elderly care physician when:

- The patient has a systolic blood pressure >200 mmHg
- The patient has a pulse of >100 beats per minute
- The patient has an irregular pulse
- A side effect of medication withdrawal is suspected

The elderly care physician will then decide: 1) continue withdrawal of medication; 2) restart medication, and fill in a (Serious) Adverse Event form

8.2.2 Serious adverse events (SAEs)

A serious adverse event is any untoward medical occurrence or effect that

- results in death;
- is life threatening (at the time of the event);
- requires hospitalisation or prolongation of existing inpatients' hospitalisation;
- results in persistent or significant disability or incapacity;
- is a congenital anomaly or birth defect; or
- any other important medical event that did not result in any of the outcomes listed above due to medical or surgical intervention but could have been based upon appropriate judgement by the investigator.

Serious adverse events will be defined as death, myocardial infarction, stroke, transient ischemic attack, or any non-elective hospitalization between randomization and the end of follow-up. An elective hospital admission will not be considered as a serious adverse event.

The investigator will report all SAEs to the sponsor without undue delay after obtaining knowledge of the events.

The sponsor will report the SAEs through the web portal *ToetsingOnline* to the accredited METC that approved the protocol, within 7 days of first knowledge for SAEs that result in death or are life threatening followed by a period of maximum of 8 days to complete the initial preliminary report. All other SAEs will be reported within a period of maximum 15 days after the sponsor has first knowledge of the serious adverse events.

8.2.3 Suspected unexpected serious adverse reactions (SUSARs)

Not applicable

8.3 Annual safety report

Not applicable

8.4 Follow-up of adverse events

All AEs will be followed until they have abated, or until a stable situation has been reached. Depending on the event, follow up may require additional tests or medical procedures as indicated, and/or referral to the general physician or a medical specialist.
SAEs need to be reported till end of study within the Netherlands, as defined in the protocol.

8.5 Data Safety Monitoring Board (DSMB)

The Danton DSMB consisting of 3 members (including clinicians and one statistician) will be installed for monitoring of the safety data (cardiovascular events). Members will be:

- Prof.dr J.W. Jukema (LUMC-cardiology, chair),
- Prof.dr W.A. van Gool (AMC-neurology, clinical expert)
- Dr. ir. N van Geloven (LUMC-department of Statistics)

Serious adverse events will be defined as death, myocardial infarction, stroke, transient ischemic attack, or any non-elective hospitalization between randomization and the end of follow-up. These adverse events will be closely monitored by the DSMB. No interim analyses for efficacy or futility will be performed.

A DSMB rapport will be send to the board after the completion of the 4 months measurements of the first 50 participants and after each additional 100 participants. This DSMB rapport will include information on number of adverse events and mean increase in blood pressure over time according to treatment allocation, number of cumulative randomisations, and (other) reasons for withdrawals/exclusions after randomisation. Possible differences between groups will be evaluated by using standard statistical

techniques. Based on these results, the DSMB will advise the principal investigators on continuing, changing or stopping the trial.

The advice(s) of the DSMB will only be sent to the sponsor of the study. Should the sponsor decide not to fully implement the advice of the DSMB, the sponsor will send the advice to the reviewing METC, including a note to substantiate why (part of) the advice of the DSMB will not be followed.

9. STATISTICAL ANALYSIS

Baseline variables will be described using descriptive statistics, using both categorical (e.g. men/women) and continuous variables (e.g. systolic blood pressure). Baseline variables showing relevant imbalance between intervention and control group will be used to adjust for in a sensitivity analysis. We will calculate 95% confidence intervals (CI) and use two-sided alpha of 0.05 to test significance. Missing data will be imputed.

9.1 Primary and secondary study parameter(s)

In the analysis of the primary outcome measure, we will employ the intention-to-treat approach. Moreover, all secondary outcome measures will also be analysed with
the intention-to-treat approach. For etiological research questions, a per protocol analysis will be employed. Descriptive, univariate and multivariate analyses will be used for comparison of the control and intervention arm.

9.2 Other study parameters

Economic evaluation

The economic evaluation will be a cost-utility analysis from healthcare perspective, with a 8-months time horizon. Use of medication and other health care use will be measured from the nursing home files and pharmacy records (including training, specialist consultations, hospitalization, lab testing, physical therapy. Healthcare use will be valued using Dutch reference prices (Zorginstituut Nederland, 2015), with nursing home care proportional to the care dependency scale (Van den Hout et al. 2014).

The possible impact of the intervention on quality of life will be estimated using the EQ-5D. Possible differences in survival will be corrected for, by assuming identical survival in both groups. Thus, QALYs will only differ due to a difference in quality of life. No baseline corrections will be performed, except for relevant baseline variables that statistically significantly differ. Using net-benefit analysis and multiple imputation, costs will be related to QALYs and presented in cost-effectiveness acceptability curves.

9.3 Interim analysis (if applicable)

Not applicable.

10. ETHICAL CONSIDERATIONS

10.1 Regulation statement

The study will be conducted according to the principles of the Declaration of Helsinki (amended most recently in October 2013), in accordance with the Medical Research Involving Human Subjects Act (WMO), the Guideline for Good Clinical Practice (May 1996), and in full conformity to any applicable state or local regulations.

10.2 Recruitment and consent

Subjects will be recruited from the UNC-ZH. Eligible residents and their legal representatives will be selected by the treating elderly care physician, after which the legal representative receives the Patient Information letter, that explains the purpose and procedures of the proposed study, the tests and questionnaires required and possible hazards that may be involved.

The legal representative will be asked to return the consent form to the researchers by mail, either with a consent, or with refusal for the patient to participate.

If the legal representative sends his/her consent, the researcher or research nurse will contact the legal representative by telephone to assure the right person has signed the form and to answer questions if necessary. If the researcher or research nurse has assured herself, she will sign the consent form as well. A copy will be retained by each legal representative and added to the medical record of the patient in the nursing home. Thereafter the resident will be enrolled in the study. Of all subjects enrolled in the study, a case report form is kept.

Participation is completely voluntarily and participants will be neither pressured nor be offered money for their participation. Participants are able to stop their participation at any time without adverse consequences, and will be explicitly told so.

10.3 Objection by minors or incapacitated subjects (if applicable)

Our research involves participants who can be considered (partially) incompetent to decide whether or not to participate in the study. In the Netherlands it is possible to carry out research with incompetent participants if there is no other group of participants in which the research question can be answered and if there is a chance that participation in the study may benefit the research subject (art. 4 lid 1 WMO). We firmly believe that this is the case in our project, since this is a study specifically dedicated to prevent or relieve NPS in dementia patients. This research question cannot be answered without the participation of subjects belonging to the group in question

Of course, every care will be taken in the informed consent procedure to explain the study in a way that is understandable for the older person involved. Also we will strive to involve

the legal representative, who might act as proxy decision maker, both at the beginning and during the study. During the study well-being of incompetent patients and the willingness to participate, as shown in reactions of incompetent patients, is taken as an important criterion for the decision whether or not to continue. This is in line with the DANTE study Leiden.

We will act in line with the CCMO code of conduct of incapacitated elderly (http://www.ccmo.nl/attachments/files/code-of-conduct-incapacitated-elderly.pdf).
If the participants objects/refuses repeatedly, he/she will be withdrawn from the study.

10.4 Benefits and risks assessment, group relatedness

Nursing home residents with dementia have a high chance of developing NPS and experiencing it negative consequences. NPS severely affect quality of life and general daily functioning, hamper optimal care, are a burden for carers and are the main reason for institutionalisation. Given the future rise in the number of older people with dementia and NPS in our society, the impact of this trail will be substantial when this trial demonstrates that NPS can be alleviated and quality of life can be improved by discontinuation of antihypertensive treatment. Since NPS hamper optimal care and are a serious burden for caregivers, this study will not only have an impact on dementia patients, but also on caregivers and nursing staff.

Patients in both study arms will continue to receive blood pressure measurements during follow-up after a stable blood pressure has been reached. This will be done for safety reasons in the intervention arm and to make both study arms as similar as possible also in the control arm. Patients in the intervention arm will be put on their original antihypertensive medication when diastolic blood pressure exceeds 120 mmHg or systolic blood pressure exceeds 200 mmHg (180 mmHg for participants with diabetes mellitus or those who had had a cardiovascular event >12 months ago) or an increase in systolic blood pressure of 60 mmHg or greater relative to baseline. Moreover, all cardiovascular events during the study will be closely monitored to prevent an increase in cardiovascular events in the intervention group. A Data Safety and Monitoring Board (DSMB) will be installed for monitoring of the safety data (cardiovascular events). The risks will not outweigh the benefits stated above. This study will not interfere with standard care, diagnostics and treatment (other than antihypertensive treatment) for patients with dementia.

10.5 Compensation for injury

Participants will be insured by a no-fault insurance of the LUMC. The sponsor/investigator has a liability insurance which is in accordance with article 7 of the WMO.

The sponsor (also) has an insurance which is in accordance with the legal requirements in the Netherlands (Article 7 WMO). This insurance provides cover for damage to research subjects through injury or death caused by the study. The insurance applies to the damage that becomes apparent during the study or within 4 years after the end of the study.

10.6 Incentives (if applicable)

Not applicable.

11. ADMINISTRATIVE ASPECTS, MONITORING AND PUBLICATION

11.1 Handling and storage of data and documents

The investigators will ensure that the participant's anonymity is maintained. In order to ensure privacy, all participant data will be coded and these codes will be used in further data analysis. Only the principal investigators will have access to the key of these codes and to the original documents. All materials and documentation with the investigation number allocated to the participant, and the listing containing the participants' name and allocation numbers are kept in strict confidence by the principle investigator. The retention of the participant's identification codes must and will be arranged by the principal investigator for at least 15 years after completion of the study. Other source data (copy of the protocol, case report forms, reports of test results, records of informed consent and other documents preparing to the conduct of the study) must be kept for at least 10 years and will be archived on an external hard disk.

In the patient information form (PIF), study participants (and their legal representatives) are informed who has access to their personal data and how, where and how long their personal data are stored. Furthermore, contact details of the local Data Protection Officer and a link to both the online privacy statement of the investigating institution and the website of the Dutch Data Protection Authority are mentioned in this document. In addition the PIF emphasizes that the consent to the use of personal data can be withdrawn at any time.

The handling of personal data complies with the European General Data Protection Regulation.

11.2 Monitoring and Quality Assurance

An external monitor will check quality of the study during regulated visits. A monitor plan will be made according to the estimated risk, to set down clear tasks.

11.3 Amendments

Amendments are changes made to the research after a favourable opinion by the accredited METC has been given. All amendments will be notified to the METC that gave a favourable opinion.

Non-substantial amendments will not be notified to the accredited METC and the competent authority, but will be recorded and filed by the sponsor.

11.4 Annual progress report

The sponsor/investigator will submit a summary of the progress of the trial to the accredited METC once a year. Information will be provided on the date of inclusion of the first subject, numbers of subjects included and numbers of subjects that have completed the trial, serious adverse events/ serious adverse reactions, other problems, and amendments.

11.5 Temporary halt and (prematurely) end of study report

The investigator/sponsor will notify the accredited METC of the end of the study within a period of 8 weeks. The end of the study is defined as the last patient's last visit.

The sponsor will notify the METC immediately of a temporary halt of the study, including the reason of such an action.

In case the study is ended prematurely, the sponsor will notify the accredited METC within 15 days, including the reasons for the premature termination.

Within one year after the end of the study, the investigator/sponsor will submit a final study report with the results of the study, including any publications/abstracts of the study, to the accredited METC.

11.6 Public disclosure and publication policy

Before the first patient is recruited, the investigator will register the trial in the trial register. The identity of the participants will not be disclosed in any way in study publications. All results will be published in peer-reviewed national and international medical journals and presented on international conferences and congresses. We also plan to publish several results in easily accessible Dutch medical journals, such as Nederlands Tijdschrift voor Geneeskunde, Tijdschrift voor Gerontologie en Geriatrie, Tijdschrift voor Ouderengeneeskunde and the Tijdschrift voor Psychiatrie. The results will also be made available through the regular information channels of the University Network for the care sector in South-Holland (UNC-ZH). Regarding the content of the publications, no agreements are or will be made with others than the participating departments of the LUMC.

Sharing data

Wherever possible individual patient data will be shared anonymously, but only after the main publication, as requested by the research program Memorabel. TOPICS-MDS will be used for both the participants and the caregiver if feasible, and appropriate. These data can be used to compare with other study populations (Lutomski et al, 2013). The legal representative will be informed about this data sharing. A Second Patient Information letter that explains the purpose and procedure of the data sharing study and a consent form will be handed or send to the legal representative with a return envelope. A signed copy will be retained by each legal representative and the received signed consent form will be added to the participant file of the patient in the research centre.

12. STRUCTURED RISK ANALYSIS

Not applicable.

13. REFERENCES

Ballard CG, Margallo-Lana M, Fossey J, et al. A 1-year follow-up study of behavioral and psychological symptoms in dementia among people in care environments. J Clin Psychiatry 2001;62(8):631-6.

Bassiony MM, Steinberg MS, Warren A, et al. Delusions and hallucinations in Alzheimer's disease: prevalence and clinical correlates. Int J Geriatr Psychiatry 2000;15(2):99-107.

Beckett NS, et al. Treatment of hypertension in patients 80 years of age or older. N Engl J Med. 2008 May 1;358(18):1887-98.

Benetos A, Labat C, Rossignol P, et al. Treatment With Multiple Blood Pressure Medications, Achieved Blood Pressure, and Mortality in Older Nursing Home Residents: The PARTAGE Study. JAMA Intern Med 2015;175(6):989-95.

Benetos A, Bulpitt CJ, Petrovic M, et al. An Expert Opinion From the European Society of Hypertension-European Union Geriatric Medicine Society Working Group on the Management of Hypertension in Very Old, Frail Subjects. Management of Hypertension in Very Old, Frail Subjects. Hypertension 2016;67(5):820-25.

Benoit M, Dygai I, Migneco O, et al. Behavioral and psychological symptoms in Alzheimer's disease. Relation between apathy and regional cerebral perfusion. Dement Geriatr Cogn Disord 1999;10(6):511-7.

Briesacher BA, Limcangco MR, Simoni-Wastila L, et al. The quality of antipsychotic drug prescribing in nursing homes. Arch Intern Med 2005;165(11):1280-5.

Brouwer WB, Van Exel NJ, Van Gorp B, et al. The CarerQol instrument: a new instrument to measure care-related quality of life of informal caregivers for use in economic evaluations. Qual Life Res 2006;15(6):1005–21.

Caljouw MA, van den Hout WB, Putter H, Achterberg WP, Cools HJ, Gussekloo J. Effectiveness of cranberry capsules to prevent urinary tract infections in vulnerable older persons: a double-blind randomized placebo-controlled trial in long-term care facilities. J Am Geriatr Soc. 2014 Jan;62(1):103-10.

Craig AH, Cummings JL, Fairbanks L, et al. Cerebral blood flow correlates of apathy in Alzheimer disease. Arch Neurol 1996;53(11):1116-20.

Cummings JL, Mega M, Gray K, Rosenberg-Thompson S, Carusi DA, Gornbein J. The Neuropsychiatric Inventory: comprehensive assessment of psychopathology in dementia. Neurology. 1994;44(12):2308–14.

Cummings, J.L., *The Neuropsychiatric Inventory: assessing psychopathology in dementia patients.* Neurology, 1997. **48** (5 Suppl 6): p. S10-6.

Dichter, M.N., et al., *Measuring the quality of life in mild to very severe dementia:testing the inter-rater and intra-rater reliability of the German version of the QUALIDEM.* Int Psychogeriatr, 2014. **26**(5): p. 825-36.

van Dijk KN, de Vries CS, van den Berg PB, et al. Drug utilisation in Dutch nursing homes. Eur J Clin Pharmacol 2000;55(10):765-71.

Dijkstra A, Sipsma D, Dassen T. Predictors of care dependency in Alzheimer’s disease after a two-year period. Int J Nurs Stud 1999;36:487–95.

Dijkstra, A., et al., *An international psychometric testing of the care dependency scale.* J Adv Nurs, 2000. **31**(4): p. 944-52.

Dijkstra, A., et al., *Further psychometric testing of the Dutch Care Dependency Scale on two different patient groups.* Int J Nurs Pract, 2002. **8**(6): p. 305-14.

Dijkstra, A., et al., Diagnostic accuracy of the care dependency scale. J Adv Nurs, 2005; 50: 410-6.

Dosa D, Intrator O, McNicoll L, et al. Preliminary derivation of a Nursing Home Confusion Assessment Method based on data from the Minimum Data Set. J Am Geriatr Soc. 2007;55(7):1099-105.

EuroQol Group. EuroQol-a new facility for the measurement of health-related quality of life. Health Policy 1990;16:199–208.

Ettema TP, Dröes RM, de Lange J, et al. QUALIDEM: development and evaluation of a dementia specific quality of life instrument--validation. Int J Geriatr Psychiatry. 2007;22(5):424–30.

Ettema TP, Dröes RM, de Lange J, et al. QUALIDEM: development and evaluation of a dementia specific quality of life instrument. Scalability, reliability and internal structure. Int J Geriatr Psychiatry. 2007;22(6):549–56.

Hartmaier SL, Sloane PD, Guess HA, et al. Validation of the Minimum Data Set Cognitive Performance Scale: agreement with the Mini-Mental State Examination. J Gerontol A Biol Sci Med Sci. 1995;50(2):M128-33.

Hirono N, Mori E, Ishii K, et al. Frontal lobe hypometabolism and depression in Alzheimer's disease. Neurology 1998;50(2):380-3.

Hirono N, Mega MS, Dinov ID, et al. Left frontotemporal hypoperfusion is associated with aggression in patients with dementia.Arch Neurol 2000;57(6):861-6.

Hoefman RJ, van Exel NJ, Looren de Jong S, et al. A new test of the construct validity of the CarerQol instrument: measuring the impact of informal care giving. Qual Life Res 2011;20: 875e887.

Hoefman RJ, van Exel NJ, Foets M, et al. Sustained informal care: The feasibility, construct validity and test-retest reliability of the CarerQol-instrument to measure the impact of informal care in long-term care. Aging Ment Health. 2011;15(8):1018-27.

Hurley AC, Volicer BJ, Hanrahan PA, Houde S, Volicer L. Assessment of discomfort in advanced Alzheimer patients. Res Nurs Health 1992;15:369-77.

Iadecola C. The pathobiology of vascular dementia. Neuron 2013;80(4):844-66.

van Iersel MB, Zuidema SU, Koopmans RT, et al. Antipsychotics for behavioural and psychological problems in elderly people with dementia: a systematic review of adverse events. Drugs Aging 2005;22(10):845-58.

James PA, Oparil S, Carter BL, et al. 2014 evidence-based guideline for the management of high blood pressure in adults: report from the panel members appointed to the Eighth Joint National Committee (JNC 8). JAMA 2014;311(5):507-20

Kat, MG, et al., [Neuropsychiatric symptoms of dementia: psychometric aspects of the Dutch Neuropsychiatric Inventory (NPI)]. Tijdschr Gerontol Geriatr 2002;33:150-5.

Katz, S., et al., Studies of illness in the aged. The index of ADL: A standardized measure of biological and psychosocial function. JAMA 1963;185:914-9.

Katz S, Down TD, Cash HR, et al. Progress in the Development of the Index of ADL. Gerontologist 1970;10:20–30.

Koopmans RT, van der Borgh JP, Bor JH, et al. Increase in drug use after admission to Dutch nursing homes. Pharm World Sci 2003;25(1):30-4.

Krabbe PF, Stouthard ME, Essink-Bot ML, Bonsel GJ. The effect of adding a cognitive dimension to the EuroQol multiattribute healthstatus classification system. J Clin Epidemiol 1999; 52: 293-301.

Laan, W., et al., Validity and reliability of the Katz-15 scale to measure unfavourable health outcomes in community-dwelling older people. J Nutr Health Aging, 2014; 18(9): 848-54.

Leontjevas, R., Evers-Stephan, A., Smalbrugge, M., Pot, A. M., Thewissen, V., Gerritsen, D. L., & Koopmans, R. T. (2012). A comparative validation of the abbreviated apathy evaluation scale (AES-10) with the neuropsychiatric inventory apathy subscale against diagnostic criteria of apathy. Journal of the American Medical Directors Association, 13(3), 308-e1.

Lueken, U., Seidl, U., Völker, L., Schweiger, E., Kruse, A., & Schröder, J. (2007). Development of a short version of the Apathy Evaluation Scale specifically adapted for demented nursing home residents. The American journal of geriatric psychiatry, 15(5), 376-385.

Lutomski JE, Baars MA, Schalk BW, et al. The Development of the Older Persons and Informal Caregivers Survey Minimum DataSet (TOPICS-MDS): A Large-Scale Data Sharing Initiative. PLoS One 2013;8:e81673.

Marshall GA, Monserratt L, Harwood D, et al. Positron emission tomography metabolic correlates of apathy in Alzheimer disease. Arch Neurol 2007;64(7):1015-20.

Matsushita K, Kuriyama Y, Nagatsuka K, et al. Periventricular white matter lucency and cerebral blood flow autoregulation in hypertensive patients. Hypertension 1994;23(5):565-8.

Mega MS, Lee L, Dinov ID, et al. Cerebral correlates of psychotic symptoms in Alzheimer's disease. J Neurol Neurosurg Psychiatry 2000;69(2):167-71.

Melis, R.J., et al., The influence of multimorbidity on clinical progression of dementia in a population-based cohort. PLoS One 2013; 8: p. e84014.

Moonen JEF and Foster-Dingley JC, de Ruijter W, et al. Effect of discontinuation of antihypertensive treatment in elderly people on cognitive functioning-the DANTE Study Leiden-: A Randomized Clinical Trial. JAMA Intern Med 2015;175(10):1622-30.

Moonen JEF and Bertens AS, Foster-Dingley JC, et al. Lower blood pressure and apathy coincide in older persons with poorer functional ability. J Am Geriatr Soc. 2015;63(1):112-7.

Morris JN, Fries BE, Mehr DR, et al. MDS Cognitive Performance Scale. J Gerontol Med Sci 1994;49:M174-82.

Muller M, Smulders YM, de Leeuw PW, et al. Treatment of hypertension in the oldest old: a critical role for frailty? Hypertension. 2014;63(3):433-41

Oeppen J, Vaupel JW. Demography. Broken limits to life expectancy. Science 2002;296:1029–31.

Oostenbrink JB, Bouwmans CAM, Koopmanschap MA, Rutten FFH. Handleiding voor onderzoek, methoden en standaard kostprijzen voor economische evaluaties in de gezondheidszorg. College voor zorgverzekeringen. Geactualiseerde versie 2004.

O'Shea E, O'Reilly S. The economic and social costs of dementia in Ireland. Int J Geriatr Psychiat 2000;15:208–18.

Ott A, Breteler MM, van Harskamp F, et al. Incidence and risk of dementia. The Rotterdam Study. Am J Epidemiol 1998;147:574–80.

Reisberg B, Ferris SH, de Leon MJ, et al. The Global Deterioration Scale for assessment of primary degenerative dementia. Am J Psychiatry. 1982;139:1136–1139.

Ruitenberg A, den Heijer T, Bakker SL, et al. Cerebral hypoperfusion and clinical onset of dementia: the Rotterdam Study. Ann. Neurol 2005; 57(6):789–94

Sabayan B, Oleksik AM, Maier AB, et al. High blood pressure and resilience to physical and cognitive decline in the oldest old: the Leiden 85-plus Study. J Am Geriatr Soc 2012;60(11):2014-9

Steinberg M, Corcoran C, Tschanz JT, et al. Risk factors for neuropsychiatric symptoms in dementia: the Cache County Study. Int J Geriatr Psychiatry 2006;21(9):824-30.

Steinberg M, Hess K, Corcoran C, et al. Vascular risk factors and neuropsychiatric symptoms in Alzheimer's disease: the Cache County Study. Int J Geriatr Psychiatry 2014;29(2):153-9.

Van den Hout WB, Caljouw MA, Putter H, Cools HJ, Gussekloo J. Cost-effectiveness of cranberry capsules to prevent urinary tract infection in long-term care facilities: economic evaluation with a randomized controlled trial. J Am Geriatr Soc. 2014; 62:111-6.

van der Steen JT, Ooms ME, van der Wal G, Ribbe MW. Measuring discomfort in patients with dementia. Validity of a Dutch version of the Discomfort Scale--dementia of Alzheimer type (DS-DAT)]. [Article in Dutch] Tijdschr Gerontol Geriatr. 2002 Dec;33(6):257-63.

Wetzels RB, Zuidema SU, de Jonghe JF, et al. Course of neuropsychiatric symptoms in residents with dementia in nursing homes over 2-year period. Am J Geriatr Psychiatry 2010;18(12):1054-65.

Williamson JD, et al. Intensive vs Standard Blood Pressure Control and Cardiovascular Disease Outcomes in Adults Aged ≥75 Years: A Randomized Clinical Trial. JAMA. 2016 Jun 28;315:2673-82.

Willie CK, Tzeng YC, Fisher JA, et al. Integrative regulation of human brain blood flow. J Physiol. 2014;592(5):841-59

Zorginstituut Nederland. Richtlijn voor economische evaluaties in de gezondheidszorg. Diemen, 2015.

Zuidema, S.U., et al., Neuropsychiatric symptoms in nursing home patients: factor structure invariance of the Dutch nursing home version of the neuropsychiatric inventory in different stages of dementia. Dement Geriatr Cogn Disord, 2007. 24(3): p. 169-76.

Appendix 1: schedule of discontinuation for Danton participants

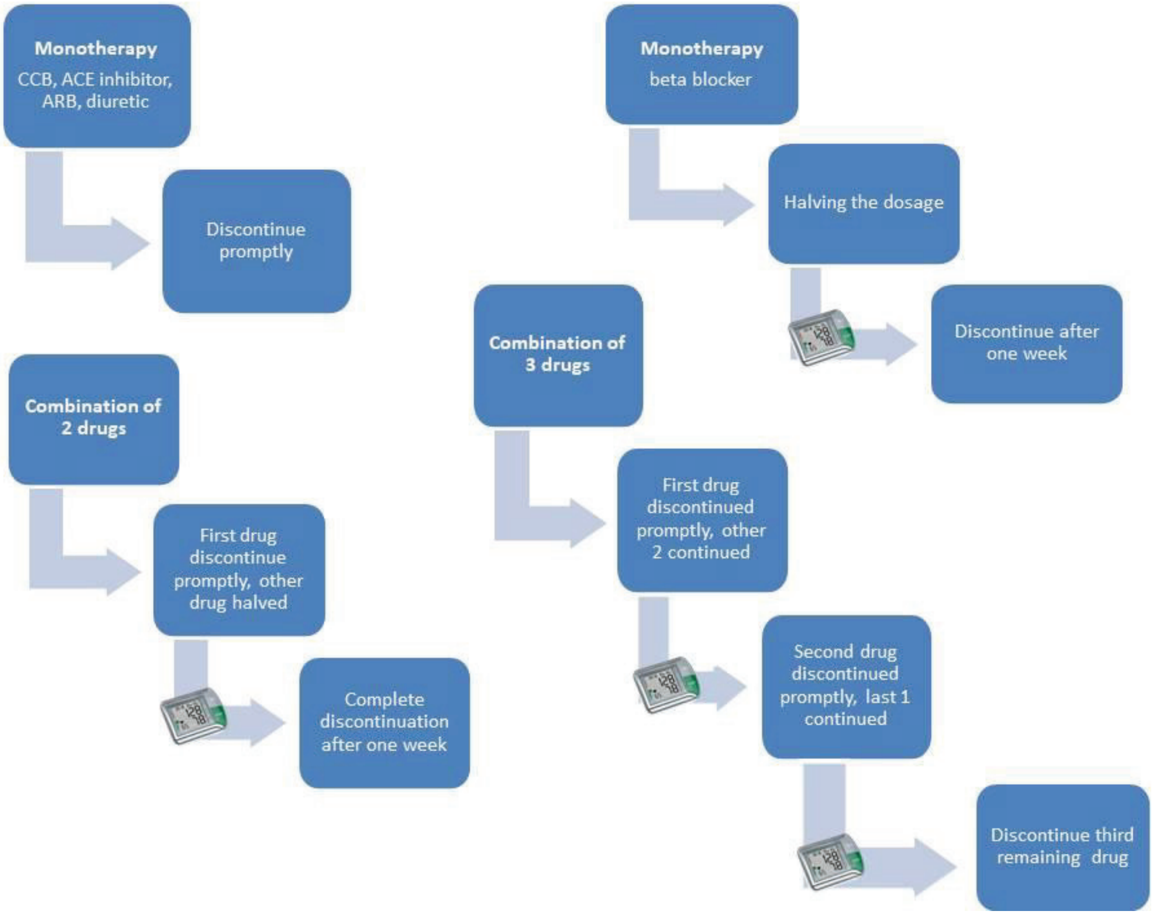

Appendix 2: overview of information collection in Danton Study

Appendix 2. Overview of information to collect in Danton study (version 30-3-2018)

	T0	T4	T8	Information from	Information collected by	Time for nursing staff
**Baseline characteristics**						
Sociodemographics participant	x			Informal caregiver	Research nurse	
Medical history at baseline (including cardiovascular status)	x			Medical record	Research nurse	
Medication history	x			Medical record	Research nurse	
Reisberg Geriatric Dementia Scale	x			Nursing staff	Research nurse	5 min
**Co-Primary outcomes**						
NPI-NH	x	x	x	Nursing staff	Research nurse	15 min
Qualidem	x	x	X	Nursing staff	Research nurse	5 min
EQ5-D+C	x	x	x	Nursing staff	Research nurse	< 5 min
**Secundary outcomes**						
**Participants**						
Blood pressure + orthostatic hypotension (sitting 2x and 3x standing, digital measurements)	x	x	x	Participant	Research nurse	10 min
The 7-category Minimum Data Set Cognitive Performance Scale	x	x	x	Participant	Research nurse	10 min
Katz				Nursing staff	Research nurse	10 min
Care dependency Scale	x	x	x	Nursing staff	Research nurse	8 min
DS-DAT (comfort scale)	x	x	x	Nursing staff	Research nurse	10 min
NH-CAM (delirium scale)	x	x	x	Nursing staff	Research nurse	5 min
AES-10	x	x	x	Nursing staff	Research nurse	5 min
**Informal Care giver outcomes**						
Demografics + relation to dementia patient	x			Informal caregiver	Digital or paper	5 min
Hours of informal care per week	x	x	x	Informal caregiver	Digital or paper	5 min
CarerQoL-7D + VAS	x	x	x	Informal caregiver	Digital or paper	10 min
**Nursing staff outcomes**						
CAREgiver occupational distress (NPI-NH subscale)	x	x	x	Nursing staff	Research nurse	
**Specifics from medical reports**						
NPS reported	x	x	X	Medical record	Research nurse	
Use of Psychotropic medication	x	x	X	Medical record	Research nurse	
Use of psychosocial interventions for NPS	x	x	X	Medical record	Research nurse	
Incident falls	x	x	x	Medical record	Research nurse	
Medical care costs (including medication, interventions, hospitalisations)	x	x	x	Medical record	Research nurse	
Cardiovascular events	x	x	X	Medical record	Research nurse	
**Information on intervention**						
Weekly blood pressure measurement by nursing staff		x	x	Medical record	Research nurse	
Medical information about deprescribing by treating doctor		x	x	Medical record	Research nurse	
