# Supplementary material for: Effects of the discontinuation of antihypertensive treatment on neuropsychiatric symptoms and quality of life in nursing home residents with dementia (DANTON): a multicentre, open-label, blinded-outcome, randomised controlled trial
Source: Age Ageing. 2024 Jul 6;53(7):afae133. doi: 10.1093/ageing/afae133 (PMC11227112; doi:10.1093/ageing/afae133)
Supplement: aa-24-0274-File007_afae133(1) [file aa-24-0274-file007_afae133(1).pdf]

DANTON Statistical Analysis Plan version 1.0

STUDY TITLE: ***D***iscontinuation of ***AN***tihypertensive ***T***reatment in ***O***lder people with dementia living in a ***N***ursing home (DANTON)

NTR REGISTER ID Trial NL7365 (NTR7573)

MEC PROTOCOL NUMBER: NL65719.058.18

PROTOCOL VERSION: Version 8 (26-05-2020)

PRINCIPAL INVESTIGATORS:

Prof. dr. J. Gussekloo
Dept. of Public Health and Primary Care
Leiden University Medical Center
PO box 9600
2300 RC Leiden

Dr. R.K.E. Poortvliet
Dept. of Public Health and Primary Care
Leiden University Medical Center
PO box 9600
2300 RC Leiden

Prof. dr. W.P. Achterberg
Dept. of Public Health and Primary Care
Leiden University Medical Center
PO box 9600
2300 RC Leiden

DATE May 22nd 2022

PREPARED BY:

Prof. dr. J. Gussekloo

Prof. dr. S. le Cessie

Dr. R.K.E. Poortvliet

Drs. J.M.K. Bogaerts

GENERAL COMMENT This statistical analysis plan (SAP) was written and approved after the preliminary termination of the study intervention by the Data Safety Monitoring Board, but before the final unblinding of the data for statistical analysis.

Table of Contents

1. Protocol overview 3
2. General objectives 4
3. Study design........ 4
4. Sample Size calculation........ 7
5. Outcomes 8
6. Populations and subgroups to be analyzed........10
7. Analyses 12

1. Protocol Overview

**Protocol Title**	**D**iscontinuation of **AN**tihypertensive **T**reatment in **O**lder people with dementia living in a **N**ursing home
**Study Activation Date**	**December 17**, **2018** (first randomization of participant)
**Study Design**	Randomized open-label, single-blind controlled clinical trial
**Study Objectives**	To assess whether discontinuation of antihypertensive treatment in nursing home residents with dementia:<br>a) reduces neuropsychiatric symptoms and improves quality of life<br>b) improves general daily functioning and cognitive functioning<br>c) reduces psychotropic medication use, falls, care dependency and caregiver burden<br>d) is safe regarding cardiovascular events.
**Treatment Description**	Randomization to discontinuation (n=246) or continuation (n=246) of antihypertensive treatment during a period of 8 months. Discontinuation of antihypertensive treatment (theoretically) aimed to achieve a systolic blood pressure increase of 20 mmHg using a drug-specific discontinuation algorithm.
**Inclusion Criteria**	Residents from nursing homes could participate if they:<br>• had a diagnosis of moderate to severe dementia according to the Reisberg Global Deterioration Scale (score 5-6-7)<br>• were on antihypertensive treatment with a calcium antagonist, diuretic, ACE-inhibitor, beta-blocker or angiotensin-II-receptor blocker prescribed for hypertension<br>• had a systolic blood pressure ≤160mmHg (average of two last blood pressure measurements)
**Exclusion Criteria**	• Recent (<12 months) history of myocardial infarction, stroke or coronary reperfusion procedures (CABG/PCI)

	• Heart failure NYHA class III or IV<br>• Current angina pectoris<br>• a life-expectancy less than 4 months.
**Study Outcomes**	The co-primary outcome measures are the differences in change of scores between 0 and 4 months on the Neuropsychiatric Inventory – Nursing Homes (NPI-NH) and quality of life. Secondary outcome measures include neuropsychiatric symptoms, apathy, care dependency, cognitive function, general daily functioning, care-related quality of life, orthostatic hypotension, incident falls, and psychotropic medication use. Long-term effects on primary and secondary outcomes will be analyzed over 8 months. In addition, cost-effectiveness will be evaluated if applicable.

2. General objectives

The overarching aim of the DANTON Trial was to study the effects of discontinuation of antihypertensive medication in older dementia patients. We hypothesized that increasing blood pressure by discontinuation of antihypertensive treatment would reduce neuropsychiatric symptoms and improve quality of life in nursing home residents with moderate to severe dementia.

3. Study design

The trial was conducted in 32 organizations for long term care, spread over 8 out of 12 provinces of the Netherlands. The included facilities covered metropolitan, urban and rural areas, providing care for nursing home residents of all ethnic backgrounds and socio-economical classes.

After informed consent, baseline information, blood pressure and baseline measurements were gathered by the research nurse. Thereafter, patients were randomized on a 1:1 ratio to parallel discontinuation (intervention) or continuation (control) of antihypertensive treatment. Stratified block randomization was used (with

variable block sizes per nursing home organization) to ensure that intervention and control participants were equally distributed within each nursing home organization. Additional stratified block randomization was used to ensure that intervention and control patients were equally distributed according to their severity of neuropsychiatric symptoms at baseline (binary, total NPI-NH score ≤12 vs >12). Concealment of treatment allocation was ensured by a central computerized randomization procedure. Participants and caregivers were not blinded to treatment allocation. Research personnel was blinded for treatment allocation.

The elderly care physician was informed by one of the members of the research center, who was (by exception) unblinded, about the outcome of randomization. Within six weeks from randomization, discontinuation was executed and completed by the patients' own elderly care physician. Deprescribing of antihypertensive treatment was continued until all drugs were stopped or a maximum systolic blood pressure of 180 mmHg was reached (theoretical target of 20 mmHg increase in systolic blood pressure). In case an adverse event occurred (e.g. a pulse of >100 beats per minute, irregular pulse, signs of cardiac decompensation), the elderly care physician had always the freedom to stop the deprescribing protocol or restart antihypertensive treatment. During the discontinuation period of six weeks blood pressure was monitored weekly until a stable blood pressure had been reached.

At four and eight months after randomization, all participants were visited again by the research nurse for assessment of all outcome measures. Most data was collected from formal and informal caregivers/legal guardians (the latter with a questionnaire by mail). The (appointed) formal caregiver of the participant was involved in the assessments of outcomes. In circumstances when the nursing home was not accessible for external visitors (e.g. when a pandemic prevention plan was activated), the research nurse contacted the appointed formal caregiver for an assessment of all outcome measures during a telephone interview. Blood pressure measurements were in such circumstances performed by the nursing home staff (conform protocol).

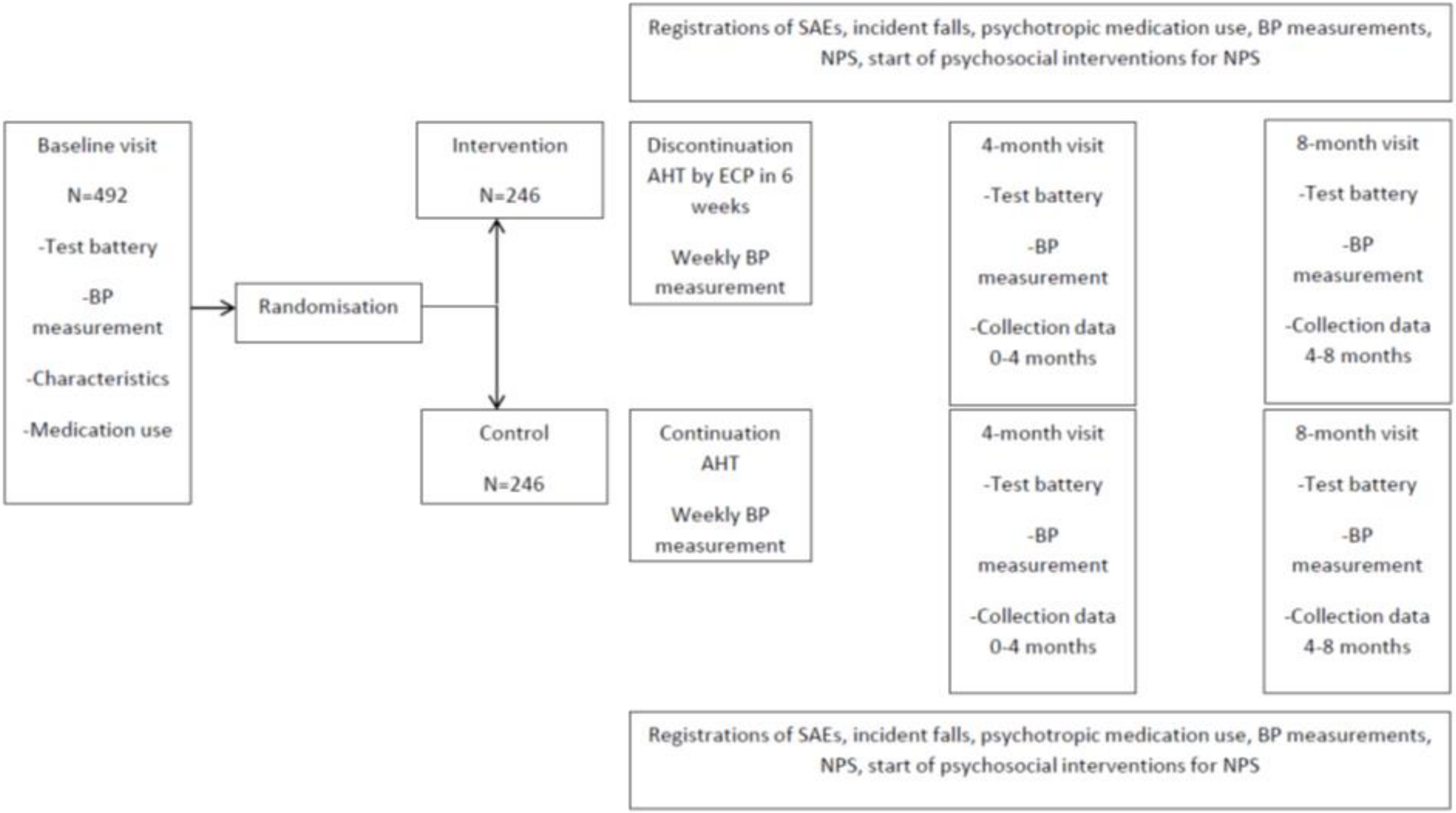

Figure 1. Flowchart describing the study design of the DANTON Trial.
BP: blood pressure. AHT: antihypertensive treatment. ECP: elderly care physician. SAE: Serious adverse events. NPS: neuropsychiatric symptoms.

4. Sample Size calculation

The sample size calculation is based on the two primary outcome measures, being the change in:

1) **neuropsychiatric symptoms (measured with the NPI-NH)**

and

2) **quality of life (measured with the Qualidem)**

between baseline and follow-up at 4 months.

For both calculations we used an alpha of 0.05 and a power of 0.9. With a clinically relevant difference of 4 points and an estimated standard deviation of 11 on the **NPI-NH** between the two groups, 159 participants are required per trial group.

To find a clinically relevant change of 10% on the short version of the **Qualidem**, with an estimated mean of 70 and an SD of 13 (linearly transformed version), a total number of 72 participants are needed in each trial group.

$$k = \frac{n_2}{n_1} = 1$$
$$n_1 = \frac{(\sigma_1^2 + \sigma_2^2/K)(z_{1-\alpha/2} + z_{1-\beta})^2}{\Delta^2}$$
$$n_1 = \frac{(11^2 + 11^2/1)(1.96 + 1.28)^2}{4^2}$$
$$n_1 = 159$$
$$n_2 = K * n_1 = 159$$

$$k = \frac{n_2}{n_1} = 1$$
$$n_1 = \frac{(\sigma_1^2 + \sigma_2^2/K)(z_{1-\alpha/2} + z_{1-\beta})^2}{\Delta^2}$$
$$n_1 = \frac{(13^2 + 13^2/1)(1.96 + 1.28)^2}{7^2}$$
$$n_1 = 72$$
$$n_2 = K * n_1 = 72$$

With an estimated 35% dropout between inclusion and assessment of the two primary outcome measures, we planned to include a total number of 246 participants in each arm of the trial.

5. Outcomes

a. Primary outcomes

The co-primary outcomes of this study are neuropsychiatric symptoms in various domains measured with the **Neuropsychiatric Inventory–Nursing Homes (NPI-NH)** and quality of life measured with **Qualidem**.

b. Secondary outcomes

Questionnaires:

- Discomfort/additional Quality of life (**DS-DAT)**
- Additional Quality of life (**EQ-5D and EQ-5D+C)**
- General daily functioning (**Katz Index)**
- Care dependency (**Care Dependency Scale**)
- Cognitive function (**7-category MDS-CPS**)
- Formal caregiver burden (**NPI-NH Subscale**)
- Informal caregiver burden (**CarerQoL-7D**)
- Presence of delirium (**Confusion Assessment Method**)

Secondary outcomes continuously recorded during study period:

- Concomitant psychotropic medication use (**from medication overview**)
- Number of falls (**collected from electronic medical records [EMR]**)

Additional information:

- Deprescribing information (**from medication overview**)
- Change in blood pressure (**Protocolized recorded during visits**)

Secondary outcomes not for main paper:

- Apathy (abbreviated **Apathy Evaluation Scale**)
- Orthostatic hypotension (**Protocolized recorded during visits**)
- Neuropsychiatric symptoms registered in the medical records (**from EMR**)
- Psychosocial interventions started (**from EMR**)
- Cost-effectiveness: **Only done when applicable**.

c. Safety outcomes

All serious adverse events (SAE) during the study were closely monitored and reported to Data Safety and Monitoring Board. SAE were defined as death, myocardial infarction, stroke, transient ischemic attack, or any non-elective hospitalization between randomization and the end of follow-up. Additionally, one life-treating case of diarrhea and dehydration was reported as an SAE. An elective hospital admission was not considered as an SAE.

6. Populations and subgroups to be analyzed

a. Populations

- **Intention-to-treat:**

  All randomized study participants, according to the group defined by the randomization process. These participants will form the primary population for the analyses.

- **Per Protocol:**
  - **Control participants:**

    All study participants randomized to the control group **without any change** of the prescribed antihypertensive treatment between the baseline visit and follow-up at 4 months.
  - **Intervention participants:**

    All study participants randomized to the intervention group **with any reduction** of the prescribed antihypertensive treatment between the baseline visit and follow-up at 4 months.

Since the control group comprised usual care, including necessary treatment intensification or urgent reduction of antihypertensive treatment (in context of detrimental hypotension), the predefined control group from the per protocol analysis could become too small. In case prescribed antihypertensive treatment of **more than 25% of control patients** was changed between baseline and 4 months follow-up, the control group of the per protocol analysis will consist of all study participants randomized to the control group (conform Intention-to-treat).

b. Subgroups

Only two subgroups will be analyzed. This subgroup will be analyzed using both Intention-to-treat and per protocol populations.

- **Stratification based on baseline NPI-NH score:**
  All randomized study participants having a total NPI-NH score ≤12 vs >12 at baseline visit.

- **Stratification based on baseline Qualidem score:**
  All randomized study participants having a total Qualidem score <70 vs ≥70 (linearly transformed version) at baseline visit.

7. Analyses

a. Descriptive statistics at baseline

Summary baseline variables will be presented by randomized group (intervention vs control) and overall, using both categorical and continuous variables. Normally distributed continuous data will be presented by the mean and standard deviation, but if more appropriate, by the median and interquartile range. There will be **no testing for statistical significance** between randomized groups for any baseline variable, because due to randomization, observed differences between groups will be purely due to chance.

b. Primary outcomes

In the analysis of the primary outcome, the change calculated **by subtracting the baseline score from the follow-up score at 4 months** will be the dependent variable. This change will be compared between the 2 randomization groups using a **linear regression** model including the **baseline score** of the investigated outcome as a covariate and **randomization group** as a factor (ANCOVA approach). Since we **balanced randomization** with the baseline total NPI-NH score (binary, ≤12 vs >12), we will also add a second factor, being **high or low total NPI-NH score at baseline**. And finally, because we **randomized within nursing home organizations**, we adjusted for **site effect by adding a random site effect to the model**. The estimated difference in mean change from baseline to 4 months between both randomization groups and the corresponding 95% confidence interval will be presented.

c. Secondary outcomes - questionnaires

The secondary outcomes will be analyzed using the same method as for the primary outcome, including the use of a **linear regression model** with **baseline scores** of the individual secondary outcome as a covariate (or **logistic regression** for **delirium)**, and **randomization group** and **high or low total NPI-NH score** at baseline both as factors. **Nursing home organization will be modelled as a random effect.** The change in follow-up score at 8 months will be calculated by subtracting the baseline score from the follow-up score at 8 months.

d. Secondary outcomes - continuously recorded during study period

Several secondary outcomes were continuously recorded during the study period, being in essence the number of falls and prescription of psychotropic drugs.

Number of falls (in the period of 16 weeks before the follow-up measurement) will be analyzed using a **poisson regression analysis**, including the correction for **the number of falls** at baseline (in the period of 16 weeks before baseline), **randomization group** and a **high or low total NPI-NH score** at baseline. **Nursing home organization will be modelled as a random effect.**

The prescription of psychotropic drugs will be analyzed as a comparison of the proportion of prescribed drug per class between randomization groups. These proportions will be compared between the 2 randomization groups using a **binary logistic regression** model with **prescription of the investigated drug class** at baseline, **randomization** group and **high or low total NPI-NH score** at baseline all three as a factor**. Nursing home organization will be modelled as a random effect.**

In summary, except (1) **baseline score or present/absent at baseline** of the investigated outcome, (2) **high or low total NPI-NH score at baseline** (due to the balanced randomization) and (3) **nursing home organization (site),** no additional adjustments or corrections will be used.

e. Safety outcomes (four analyses, not corrected, KM)

The number and characteristics of SAE will be summarized and split by randomization group. Kaplan-Meier plots by randomization group for the time to the first occurrence of **both all-cause SAE and all-cause mortality** will be separately produced. Time to event will be compared between groups using **log-rank** tests.

Furthermore, Kaplan-Meier plots for **cardiovascular SAE** and **cardiovascular mortality with competing risk of non-cardiovascular SAE and non-cardiovascular mortality** will be separately produced. Time to event will be

compared between groups using log-rank tests. In the safety analysis, no adjustments or corrections will be used.

f. Sensitivity analysis

Two sensitivity analyses will be performed.

First, the analysis of the primary outcomes (**NPI-NH** total score and **QUALIDEM** total score) will be repeated with adjustments for (1) **sex**, (2) **history of cardiovascular diseases** and (3) baseline variables showing **relevant imbalance** between intervention and control group. We define relevant imbalance as:

- a difference greater than **the reported overall standard deviation** of the baseline variable
- a difference greater than **10% of the reported overall median** of the baseline variable

As a second sensitivity analysis, we will repeat the analysis of the primary outcome with **imputation of missing variables** (see missing data).

g. Missing Data

In the primary analysis of all outcomes we will use **complete cases**. In a sensitivity analysis (see above), we will impute, under **the missing at random assumption (measurements after death will not be imputed)**, the missing data of the primary outcomes. Missing values will be imputed using multiple imputation.

h. Used software

All analyses, except the analysis of cardiovascular SAE and death, will be performed with SPSS software (version 25.0; IBM Corp). For the analysis of cardiovascular SAE and cardiovascular death, R 4.1.2 with software package CMPRSK will be used.
